# Analysis of Diffusion Tensor Imaging Data From the UK Biobank Confirms Dosage Effect of 15q11.2 Copy Number Variation on White Matter and Shows Association With Cognition

**DOI:** 10.1016/j.biopsych.2021.02.969

**Published:** 2021-09-01

**Authors:** Ana I. Silva, George Kirov, Kimberley M. Kendall, Mathew Bracher-Smith, Lawrence S. Wilkinson, Jeremy Hall, Magnus O. Ulfarsson, G. Bragi Walters, Hreinn Stefansson, Kari Stefansson, David E.J. Linden, Xavier Caseras

**Affiliations:** a Neuroscience and Mental Health Research Institute, MRC Centre for Neuropsychiatric Genetics and Genomics, Cardiff, United Kingdom; b Cardiff University Brain Research Imaging Centre School of Psychology, Cardiff University, Cardiff, United Kingdom; c Division of Psychological Medicine and Clinical Neurosciences, School of Medicine, Cardiff University, Cardiff, United Kingdom; d School of Psychology, Cardiff University, Cardiff, United Kingdom; e School for Mental Health and Neuroscience, Department of Psychiatry and Neuropsychology, Faculty of Health, Medicine and Life Sciences, Maastricht University, Maastricht, the Netherlands; f deCODE genetics/Amgen, Reykjavik, Iceland; g Faculty of Electrical and Computer Engineering, University of Iceland, Reykjavik, Iceland; h Faculty of Medicine, University of Iceland, Reykjavik, Iceland

**Keywords:** 15q11.2 BP1-BP2, Cognition, Copy number variant, CYFIP1, Diffusion tensor imaging, Genetics

## Abstract

**Background:**

Copy number variations at the 15q11.2 BP1-BP2 locus are present in 0.5%–1.0% of the population, and the deletion is associated with several neurodevelopmental disorders. Previously, we showed a reciprocal effect of 15q11.2 copy number variation on fractional anisotropy, with widespread increases in deletion carriers. We aim to expand these findings using a larger sample of participants (*N* = 29,166) and higher resolution imaging and by examining the implications for cognitive performance.

**Methods:**

Diffusion tensor imaging measures from participants with no neurological or psychiatric diagnoses were obtained from the UK Biobank database. We compared 15q11.2 BP1-BP2 deletion (*n* = 102) and duplication (*n* = 113) carriers to a large cohort of control individuals with no neuropsychiatric copy number variants (*n* = 28,951). Additionally, we assessed how changes in white matter mediated the association between carrier status and cognitive performance.

**Results:**

Deletion carriers showed increases in fractional anisotropy in the internal capsule and cingulum and decreases in the posterior thalamic radiation compared with both duplication carriers and control subjects (who had intermediate values). Compared with control subjects, deletion carriers had lower scores across cognitive tasks, which were partly influenced by white matter. Reduced fractional anisotropy in the posterior thalamic radiation partially contributed to worse cognitive performance in deletion carriers.

**Conclusions:**

These results, together with our previous findings, provide convergent evidence for an effect of 15q11.2 BP1-BP2 on white matter microstructure, this being more pronounced in deletion carriers. Additionally, changes in white matter were found to partially mediate cognitive ability in deletion carriers, providing a link between white matter changes in 15q11.2 BP1-BP2 carriers and cognitive function.

Several copy number variants (CNVs) are associated with neurodevelopmental disorders (NDDs) in genome-wide studies, including intellectual disability, autism spectrum disorder (ASD), epilepsy, and schizophrenia (1, 2, 3). Altered white matter is common in many NDDs and has been shown to mediate core cognitive deficits in schizophrenia (4,5). However, whether alterations in white matter microstructure are associated with these CNVs and can explain—at least partly—cognitive deficits in carriers of the risk variants is not yet fully understood.

The chromosome 15q11-13 region contains five breakpoints (BPs) that can give rise to CNVs (6). Deletions and duplications at 15q11.2 BP1-BP2 are the most prevalent in humans, being present in 0.5%–1.0% of the general population (7,8). Deletions are associated with developmental and motor delays (9), as well as increased susceptibility to attention-deficit/hyperactivity disorder, ASD, schizophrenia, epilepsy (1,10), and congenital heart disease (11, 12, 13), whereas the pathogenicity of the corresponding duplication is less clear in population samples, where a significant risk for NDDs has not been established (1,3) despite its link to neurodevelopmental phenotypes in clinical samples (14,15). Carriers of the 15q11.2 BP1-BP2 deletion, unaffected by a diagnosed NDD, show lower cognitive function than noncarrier control subjects (11,16), as well as a higher prevalence of dyslexia and dyscalculia (7,17), whereas carriers of the corresponding duplication perform similarly to control subjects on many cognitive tests (16,17).

The 15q11.2 BP1-BP2 interval comprises four genes: *NIPA1*, *NIPA2*, *CYFIP1*, and *TUBGCP5* (18). These genes are expressed in the central nervous system and have been individually associated with multiple disorders: *NIPA1* with autosomal-dominant hereditary spastic paraplegia (19), *NIPA2* with childhood absence epilepsy (20), *TUBHGCP5* with attention-deficit/hyperactivity disorder and obsessive-compulsive disorder (10), and *CYFIP1* with increasing susceptibility to ASD (21) and schizophrenia (22). A recent report investigated protein-protein interactions of the four genes in this region and found that they interact with each other and that their predicted functions encompass crucial biological processes that are important for normal neuronal development, plasticity, and function (23).

Using diffusion tensor imaging (DTI), we have previously shown an association between 15q11.2 BP1-BP2 CNV dosage and altered white matter microstructure in an Icelandic sample (24). We found widespread increases of fractional anisotropy (FA) in deletion carriers relative to duplication carriers, with noncarrier control subjects showing intermediate values, and the largest effects were observed in the posterior limb of the internal capsule. The Icelandic gene pool is less heterogeneous than that of most European populations (25), facilitating the reduction of background noise caused by genetic variation (26) but arguably also raising concerns about the replicability of these findings in more genetically diverse, heterogeneous populations (27).

The aim of this study was to expand our previous findings to a more heterogeneous European population, using a bigger sample and higher resolution imaging, and to further investigate the potential mediation effects of white matter changes on the association between 15q11.2 BP1-BP2 and cognitive performance. To this end, we used a subsample of participants (∼29,000) from the UK Biobank for whom DTI-derived measures, along with genetic data, were available. Based on our previous findings, we hypothesized a dose-effect association between 15q11.2 BP1-BP2 carrier status and DTI-based white matter measures, with greater effects in deletion carriers. We also predicted white matter changes to mediate the association between this CNV and cognitive performance in deletion carriers.

## Methods and Materials

### Participants

A subsample of participants from the UK Biobank (www.ukbiobank.ac.uk) was used in this study. Ethical approval was granted by the North West Multi-Centre Ethics committee, and all subjects provided informed consent to participate in the UK Biobank project. Data were released to Cardiff University after application to the UK Biobank (project ref. 17044).

To avoid potential confounding from population stratification, we only selected participants who self-reported as white British or Irish descent and for whom white British and Irish ancestry was confirmed, using the two first principal components provided by the UK Biobank (28) (Figure S1 in Supplement 1) (73,126 participants removed). Furthermore, to avoid confounding effects of disease, only participants with no personal history—based on self-reported diagnosis from a doctor at any assessment visit or existing hospital records—of neuropsychiatric disorders (i.e., schizophrenia, psychosis, ASD, dementia, or intellectual disability) or medical/neurological conditions that could impact white matter (i.e., alcohol or other substance dependency, Parkinson’s disease, Alzheimer’s disease, multiple sclerosis, or other neurodegenerative conditions) were selected (37,176 participants removed). After applying these exclusions, 392,340 participants remained (the number of participants excluded per carrier group and condition is presented in Table S1 in Supplement 2).

### Genotyping, CNV Calling, and CNV Quality Control

DNA extraction and processing workflow are described at https://biobank.ctsu.ox.ac.uk/crystal/crystal/docs/genotyping_sample_workflow.pdf. CNV calling was performed by Kendall *et al.* (29), and quality control parameters are briefly explained in Supplemental Methods in Supplement 1. Carriers of CNVs at the 15q11.2 BP1-BP2 locus and participants with no neurodevelopmental CNVs (NoCNV) were selected. For the NoCNV group, we selected participants that carried none of the 93 CNVs (Table S2 in Supplement 2) that have previously been associated with NDDs (3,30,31). The 15q11.2 BP1-BP2 interval was manually inspected to confirm that it included the key genes within the region (Table S3 in Supplement 2). We found 1468 15q11.2 BP1-BP2 deletion carriers, 1752 duplication carriers, and 358,257 NoCNV carriers in the remaining sample after exclusions and quality control.

### DTI Data

We used standard DTI measures made available by the UK Biobank. Imaging protocols can be found in brain magnetic resonance imaging documentation (http://biobank.ctsu.ox.ac.uk/crystal/crystal/docs/brain_mri.pdf). DTI data were acquired using a multishell approach with two b-values (b = 1000 and 2000 s/mm^2^). For each diffusion-weighted shell, 50 diffusion-encoding directions were acquired. Tensor fitting utilizes b = 1000 s/mm^2^ data, leading to the generation of FA, axial diffusivity (AD), radial diffusivity (RD), and mean diffusivity (MD) maps. DTI maps were used in Tract-Based Spatial Statistics (TBSS) processing, and TBSS-derived measures were computed by averaging the skeletonized images of each DTI map within a set of 48 standard-space tract masks defined by the JHU White Matter Atlas (ICBM-DTI-81) (32).

DTI data were available for 29,978 participants in the UK Biobank; of those, 102 were 15q11.2 BP1-BP2 deletion carriers, 113 were duplication carriers, and 28,951 were NoCNV carriers. Participants were aged between 40 and 70 years, and the numbers of females and males were similar in each group. Demographic information is provided in Table 1.

**Table 1:** Demographic Characteristics of Individuals With Neuroimaging Data Available From the UK Biobank After Exclusions

	Demographic Characteristics	15q11.2 BP1-BP2 CNVs			Test Statistic^a^	*p* Value
		Deletion	NoCNV	Duplication		
UK Biobank	Total	102	28,951	113		
	Male, *n* (%)	55 (54%)	13,623 (47%)	52 (46%)	χ^2^_2_ = 1.97	.37
	Age, mean (SD); [range]	55.4 (7.3); [40–68]	54.9 (7.4); [40–70]	54.8 (7.2); [40–69]	*F*_2_ = 0.42	.5
deCODE (24)	Total	30	19	27		
	Male, *n* (%)	14 (47%)	12 (63%)	12 (44%)	χ^2^_2_ = 1.78	.41
	Age, mean (SD); [range]	42.8 (12.5); [21–65]	38.9 (10.6); [22–59]	43.5 (13.5); [22–65]	*F*_2_ = 0.026	.87

See Methods and Materials for exclusion criteria. Details from the Icelandic sample (deCODE), used in our previous study (24), are shown for comparison.

CNV, copy number variant; NoCNV, no pathogenic CNVs.

a Statistical differences in sex and age between each group (deletion, NoCNV, and duplication) were assessed using χ^2^ and analysis of variance, respectively.

To avoid the potential effect of extreme values that could have resulted from poor data quality or processing problems, outlier values of FA, AD, RD, and MD, defined as values ± 2.5 SDs from the group mean, were removed from the analyses. Outlier identification was run individually for each white matter tract and within each carrier group. The number of data points excluded per tract and carrier group are presented in Table S4 in Supplement 2. Overall, no carrier group had a significant excess of outlier data points compared with the other groups.

The mean TBSS-derived measures (FA, AD, RD, and MD) from 30 white matter tracts were considered for analyses (Table 2). These included the tracts analyzed in our previous study (24) plus other regions recently highlighted because of their association with psychiatric disorders (33).

**Table 2:** The 30 White Matter Tracts Selected for This Study and Corresponding Abbreviations

White Matter Tracts	Abbreviation
Genu Corpus Callosum	GenuCC
Body Corpus Callosum	BodyCC
Splenium Corpus Callosum	SpleniumCC
Body of the Fornix	Fornix
Corticospinal Tract—Right and Left	CST_R; CST_L
Anterior Limb of the Internal Capsule—Right and Left	ALIC_R; ALIC_L
Posterior Limb of the Internal Capsule—Right and Left	PLIC_R; PLIC_L
Anterior Corona Radiata—Right and Left	ACR_R; ACR_L
Superior Corona Radiata—Right and Left	SCR_R; SCR_L
Posterior Corona Radiata—Right and Left	PCR_R; PCR_L
Posterior Thalamic Radiation—Right and Left	PTR_R; PTR_L
Sagittal Stratum (Includes Inferior Longitudinal Fasciculus)—Right and Left	SStratum_R; SStratum_L
External Capsule—Right and Left	ExtC_R; ExtC_L
Cingulum (Cingulate Gyrus Portion)—Right and Left	Cing_CG_R; Cing_CG_L
Cingulum (Hippocampus Portion)—Right and Left	Cing_HIP_R; Cing_HIP_L
Superior Longitudinal Fasciculus—Right and Left	SLF_R; SLF_L
Uncinate Fasciculus—Right and Left	Unc_R; Unc_L

### Cognitive Data

Participants in the UK Biobank also underwent a series of cognitive tests. We evaluated the performance on seven cognitive tasks that had been performed by at least 10% of the participants: the pairs matching, reaction time, fluid intelligence, digit span, symbol digit substitution, and trail making A and B tasks. Cognitive measures were normally distributed and standardized following a previously published approach (29), detailed in Supplemental Methods in Supplement 1. All cognitive measures were transformed so that lower values represented poorer performance. Table 3 describes the sample sizes used for each task in our neuroimaging sample. Because cognitive data are available for many more participants in the UK Biobank than those with neuroimaging data, we also report an extended analysis considering the full sample in Table S6 in Supplement 2.

**Table 3:** Effects of 15q11.2 BP1-BP2 Copy Number Variation on Cognitive Performance in UK Biobank Participants for Whom DTI Data Were Available

Effects	15q11.2 BP1-BP2 CNVs			Deletion vs NoCNV			Duplication vs NoCNV			Dosage	
	Deletion, *n*	NoCNV, *n*	Duplication, *n*	Cohen’s *d* (SE)	*p* Value	*p*_FDR_	Cohen’s *d* (SE)	*p* Value	*p*_FDR_	*F* Statistic	*p* Value
Pairs Matching	102	28,599	112	−0.04 (0.10)	.63	.80	0.01 (0.10)	.88	.9	*F*_2,28805_ = 0.17	.8
Reaction Time	102	28,898	113	−0.32 (0.10)	.001	.01^a^	0.17 (0.10)	.07	.1	*F*_2,29105_ = 7.14	.0008
Fluid Intelligence	89	26,690	108	−0.32 (0.10)	.003	.01^a^	−0.04 (0.10)	.71	.8	*F*_2,26879_ = 4.33	.01
Digit Span	71	19,799	81	−0.29 (0.12)	.02	.05	−0.1 (0.12)	.38	.5	*F*_2,19943_ = 3.27	.04
Symbol Substitution	62	15,127	55	−0.36 (0.13)	.005	.02^a^	0.13 (0.13)	.33	.5	*F*_2,15236_ = 4.79	.008
Trail Making A	52	13,538	48	−0.29 (0.14)	.04	.09	−0.04 (0.14)	.80	.8	*F*_2,13630_ = 2.52	.08
Trail Making B	52	13,538	48	−0.40 (0.14)	.004	.02^a^	0.19 (0.14)	.20	.3	*F*_2,13630_ = 5.67	.003

Group differences were assessed using ANOVA followed by post hoc pairwise comparisons. Both uncorrected and FDR-corrected *p* values are shown.

ANOVA, analysis of variance; BP, breakpoint; CNV, copy number variant; FDR, false discovery rate; NoCNV, no pathogenic CNVs.

a *p* < .05 after FDR correction.

### Statistical Analyses

Statistical analyses were performed in R V3.6.3 (R Foundation). CNV group effects were examined via analysis of variance, including age, sex, and handedness as covariates. For brain measures, we also included brain size (total gray matter + white matter volumes) as a covariate. Following this, post hoc pairwise comparisons were performed to measure differences between groups (deletion vs. NoCNV, duplication vs. NoCNV, and deletion vs. duplication). We used the Benjamini-Hochberg false discovery rate (*p* < .05) to account for multiple testing (34), for a total of 360 tests (30 tracts × 4 TBSS-derived measures × 3 group comparisons) in the case of imaging data and a total of 21 tests (7 cognitive test × 3 group comparisons) in the case of cognitive data. The interactions between copy number and age and between copy number and sex were also assessed. We repeated the analyses without excluding participants with neurological/psychiatric conditions (Tables S5 and S6 in Supplement 2).

Cohen’s *d* effect sizes were calculated for the pairwise comparisons. Adjusted values for each group were used, regressing out the effects of age, sex, handedness, and brain volume using linear regression. Effect sizes across TBSS-derived measures for each comparison are shown in Figures S2–S4 in Supplement 1. Cohen classified effect sizes as negligible (*d* < 0.2), small (0.2 < *d* < 0.5), medium (0.5 < *d* <0.8), and large (*d* > 0.8) (35).

To best examine the concordance between our current findings in the UK Biobank sample and our previous findings in the Icelandic deCODE sample (24), we plotted the effect sizes from both samples using forest plots. To facilitate the comparison, we recalculated the effect sizes from the Icelandic sample using adjusted values for age, sex, and brain volume, and only white matter tracts showing group differences in either study are shown (Figures S5–S7 in Supplement 1).

Mediation analysis was performed to test the hypothesis that 15q11.2 BP1-BP2 CNV effects on cognition are mediated by white matter abnormalities. Tracts showing a significant association between FA and carrier status were considered, as well as cognitive tasks that were significantly affected in carriers when compared with NoCNV carriers. Linear regression was used to look at overall effects of white matter on cognitive tasks (including all deletion and duplication carriers). Mediation analysis was conducted using the mediation package version 4.4.7 in R (http://CRAN.R-project.org/package=mediation), which uses structural equation modeling. We report the proportion of the total effect of copy number on cognitive performance mediated by FA, with *p* values calculated through quasi-Bayesian approximation using 5000 simulations. Age, sex, handedness, and brain volume were included as covariates. False discovery rate correction was again applied to account for multiple testing, in this case accounting for 28 tests (4 cognitive measures × 7 tracts).

## Results

### Group Differences on TBSS-Derived Measures

**Figure 1:**
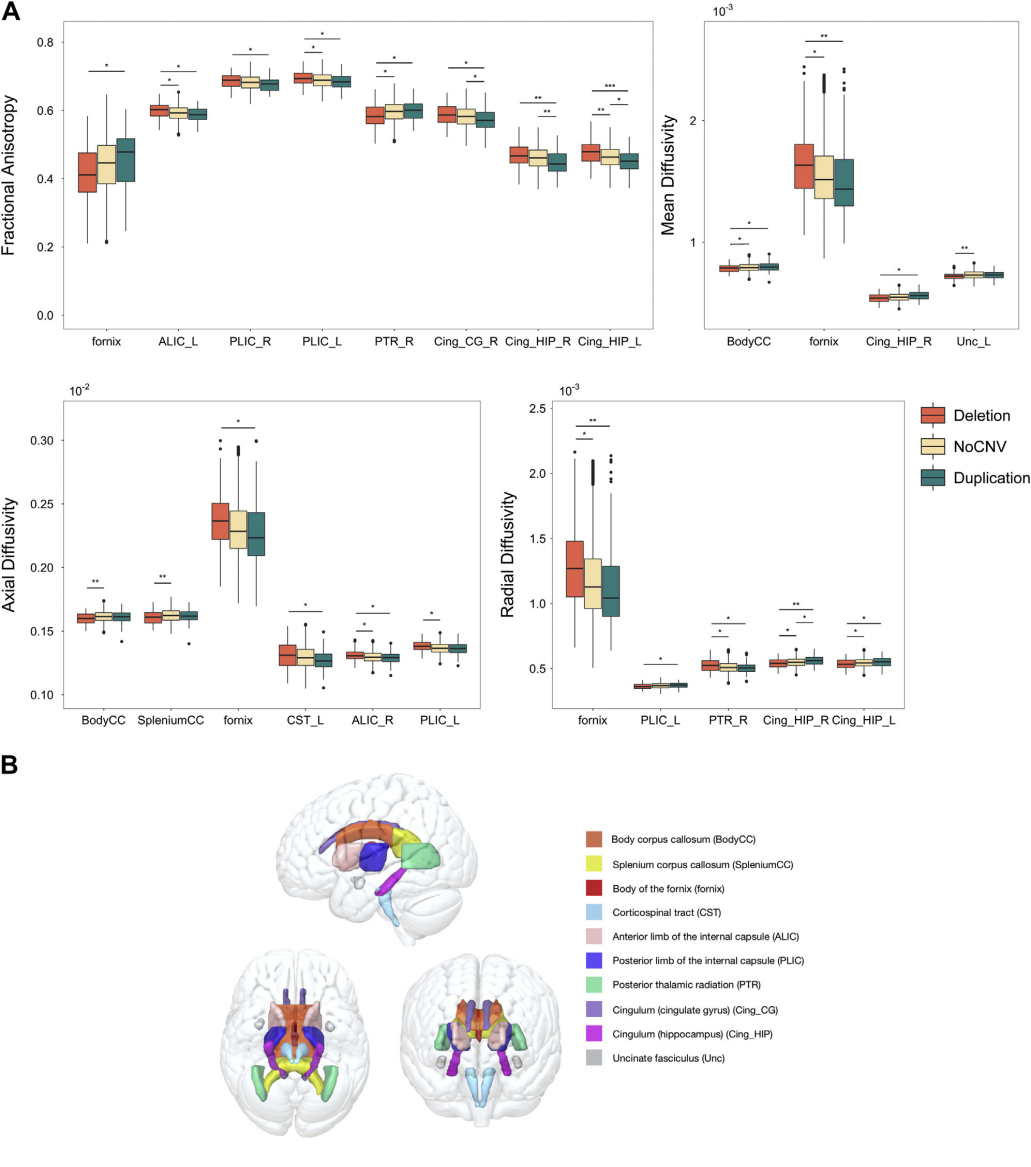
**(A)** Boxplots showing the effects of 15q11.2 BP1-BP2 copy number variation on Tract-Based Spatial Statistics–derived measures. Group differences between deletion (*n* = 102), duplication (*n* = 113), and no pathogenic copy number variant (NoCNV; *n* = 28,951) carriers were assessed with an analysis of variance followed by post hoc pairwise comparisons. Here, only white matter tracts showing significant group differences after false discovery rate correction for multiple comparisons are shown. Statistics are documented in Table S7 in Supplement 2. ∗*p* < .05, ∗∗*p* < .01, ∗∗∗*p* < .001. **(B)** Glass brain representation of the white matter regions defined by the JHU White Matter Atlas (ICBM-DTI-81).

15q11.2 BP1-BP2 deletion carriers showed increased FA relative to duplication carriers in ALIC_L, PLIC_R, PLIC_L, Cing_CG_R, Cing_HIP_R, and Cing_HIP_L and decreased FA in fornix and PTR_R (see Figure 1 and Table S7 in Supplement 2 for statistics). Deletion carriers also showed increased FA in ALIC_L, PLIC_L, and Cing_HIP_L and decreased FA in PTR_R when compared with NoCNV carriers. Additionally, deletion carriers showed significant decreases in MD in BodyCC and Unc_L and significant decreases in AD in BodyCC and SpleniumCC when compared with NoCNV carriers. 15q11.2 BP1-BP2 duplication carriers showed reduced FA in Cing_CG_R, Cing_HIP_R, and Cing_HIP_L compared with NoCNV carriers.

The effect sizes for FA were overall small (Cohen’s *d* < 0.5) when comparing carriers to noncarriers. Larger effects were found when comparing deletion to duplication carriers, these being of medium size (Cohen’s *d* > 0.5) for Cing_HIP_R, and Cing_HIP_L (Table S7 in Supplement 2), the latter showing a significant dosage effect with deletion and duplication carriers differing from NoCNV carriers in opposing directions. Diverging bar plots showing effect sizes for all 30 white matter tracts considered are shown in Figures S8–S11 in Supplement 1.

No significant CNV × age and CNV × sex interactions were found after multiple testing correction (see Supplemental Findings in Supplement 1 and Tables S8 and S9 in Supplement 2).

In the UK Biobank imaging sample, we found 4 deletion and 3 duplication carriers who carried an additional damaging CNV. We repeated the analysis removing these participants, which did not alter our results.

### Cognitive Performance in 15q11.2 CNV Carriers

In our neuroimaging sample, deletion carriers showed poorer performance on reaction time, fluid intelligence, symbol substitution, and trail making B tasks, whereas duplication carriers achieved a similar level of performance as NoCNV carriers (Table 3). This was also true when considering all participants with cognitive data (Table S6 in Supplement 2), where deletion carriers showed poorer performance in pairs matching, reaction time, fluid intelligence, digit span, symbol substitution, and trail making B tasks; duplication carriers showed poorer performance only in pairs matching task and no effects on other tasks. No significant CNV × age or CNV × sex interactions were observed for cognitive performance (see Supplemental Findings in Supplement 1 and Tables S8 and S9 in Supplement 2).

Because FA is the metric more commonly used in DTI studies and the one more widely associated with 15q11.2 BP1-BP2 carrier status here, we focused our mediation analysis only on this measure. As described above, four white matter tracts (ALIC_L, PLIC_L, PTR_R, and Cing_HIP_L) showed FA changes in deletion carriers and three tracts (Cing_CG_R, Cing_HIP_R, and Cing_HIP_L) in duplication carriers when compared with NoCNV carriers. Therefore, the effects of these tracts on fluid intelligence, reaction time, symbol substitution, and trail making B task performance were tested. FA variation in all these tracts was overall significantly associated with cognitive task performance, where increases in FA were associated with better performance (Table 4). Associations between cognitive tests and FA in regions for each CNV carrier status can be seen in Figures S14–S17 in Supplement 1. No significant CNV × FA interactions were observed in cognitive performance.

**Table 4:** Mediation Analysis Showing the Proportion of the Mediated Effect of FA on the Total Effect of 15q11.2 BP1-BP2 CNV on Reaction Time, Fluid Intelligence, Symbol Substitution, and Trail Making B

WM Tracts	Path B		Mediation Effects			
	β (SE)	*p*_FDR_	Proportion Mediated (CI)	*p* Value	*p*_FDR_	Comparison
Reaction Time						
ALIC_L	.03 (.006)	3.67 × 10^−7^^a^	−0.029 (−0.09 to −0.01)	.004	.03^a^	Del vs. NoCNV
PLIC_L	.003 (.006)	.6	−0.003 (−0.02 to 0.01)	.51	.6	
PTR_R	.03 (.006)	6.35 × 10^−5^^a^	0.024 (0.008 to 0.07)	.003	.03^a^	
Cing_HIP_L	.02 (.006)	.003^a^	−0.022 (−0.07 to −0.01)	.002	.03^a^	
Cing_CG_R	.04 (.006)	1.32 × 10^−11^^a^	−0.07 (−0.57 to 0.26)	.07	.1	Dup vs. NoCNV
Cing_HIP_R	.03 (.006)	2.6 × 10^−5^^a^	−0.052 (−0.41 to 0.2)	.07	.1	
Cing_HIP_L	.02 (.006)	.003^a^	−0.03 (−0.3 to 0.16)	.09	.2	

Fluid Intelligence						
ALIC_L	.03 (.006)	4.34 × 10^−7^^a^	−0.02 (−0.07 to 0)	.06	.1	Del vs. NoCNV
PLIC_L	.02 (.006)	.006^a^	−0.012 (−0.05 to 0)	.03	.09	
PTR_R	.05 (.006)	2.97 × 10^−12^^a^	0.044 (0.015 to 0.14)	.004	.03^a^	
Cing_HIP_L	.02 (.006)	.004^a^	−0.023 (−0.07 to −0.01)	.007	.03^a^	
Cing_CG_R	.01 (.006)	.06	0.01 (−0.59 to 0.6)	.9	.9	Dup vs. NoCNV
Cing_HIP_R	.03 (.006)	2.75 × 10^−5^^a^	0.037 (−1.33 to 1.66)	.9	.9	
Cing_HIP_L	.02 (.006)	.004^a^	−0.03 (−0.88 to 1.05)	.8	.9	

Symbol Substitution						
ALIC_L	.04 (.007)	3.65 × 10^−9^^a^	−0.05 (−0.19 to −0.01)	.009	.03^a^	Del vs. NoCNV
PLIC_L	.009 (.007)	.2	−0.006 (−0.04 to 0)	.2	.3	
PTR_R	.06 (.007)	8.94 × 10^−13^^a^	0.06 (0.02 to 0.2)	.006	.03^a^	
Cing_HIP_L	.02 (.008)	.001^a^	−0.02 (−0.09 to 0)	.01	.04^a^	
Cing_CG_R	.05 (.008)	2.7 × 10^−10^^a^	−0.13 (−2.31 to 1.6)	.3	.4	Dup vs. NoCNV
Cing_HIP_R	.02 (.008)	.04^a^	−0.03 (−0.55 to 0.35)	.3	.4	
Cing_HIP_L	.02 (.008)	.001^a^	−0.05 (−0.75 to 0.79)	.3	.4	

Trail Making B						
ALIC_L	.04 (.008)	4.11 × 10^−7^^a^	−0.06 (−0.25 to −0.02)	.01	.04^a^	Del vs. NoCNV
PLIC_L	.01 (.008)	.1	−0.01 (−0.05 to 0)	.09	.2	
PTR_R	.05 (.008)	3.65 × 10^−9^^a^	0.04 (−0.007 to 0.15)	.02	.05^a^	
Cing_HIP_L	.01 (.008)	.2	−0.01 (−0.05 to 0.01)	.2	.3	
Cing_CG_R	.03 (.008)	.0001^a^	−0.06 (−0.63 to 0.61)	.2	.3	Dup vs. NoCNV
C_HIP_R	.003 (.008)	.7	−0.003 (−0.07 to 0.04)	.7	.8	
Cing_HIP_L	.01 (.008)	.2	−0.01 (−0.28 to 0.18)	.4	.5	

Negative proportions indicate opposite signs between the mediator (FA) and the total effect (CNV effect on cognitive measure). The linear regression (Path B) shows the overall effects of white matter on cognition (including all deletion and duplication carriers). Both uncorrected and corrected (FDR) *p* values are shown.

CI, confidence interval; CNV, copy number variant; Del, deletion; Dup, duplication; FA, fractional anisotropy; FDR, false discovery rate; NoCNV, no pathogenic copy number variant; WM, white matter. See Table 2 for white matter tract abbreviations.

a *p* < .05 after FDR correction.

We found that ALIC_L, PTR_R, and Cing_HIP_L partially mediated cognitive performance in deletion carriers. Decreased FA in PTR_R was found to partially mediate cognitive performance on all four cognitive tasks, accounting for between 2.4% and 6% of the association between carrier status and cognitive performance (Table 4). Increased FA in ALIC_L in deletion carriers was associated with higher scores in reaction time, symbol substitution, and trail making B tasks, removing 2.9%, 5%, and 6% of the total effect of carrier status on each of these tasks, respectively. Similarly, increased FA in Cing_HIP_L in deletion carriers removed 2.2%, 2.3%, and 2% of the total effect of carrier status on reaction time, fluid intelligence score, and symbol substitution, respectively.

## Discussion

To our knowledge, this is the largest study to date investigating the effects of the 15q11.2 BP1-BP2 CNV on white matter microstructure, as well as the first study examining how these effects are associated with cognitive ability. Using a large sample from the UK Biobank, we found more prominent differences between deletion and NoCNV carriers than between duplication and NoCNV carriers. These results are in line with our previous findings in an Icelandic sample, also showing larger effect sizes in deletion carriers (24). Additionally, we showed that deletion carriers have poorer cognitive performance, which is partially mediated by changes in FA.

Previous results from our group using an Icelandic sample showed increased FA in deletion carriers in the left inferior longitudinal fasciculus (ILF_L), PCR_L, PTR_R, C_CG_L, ALIC_L, PLIC_R, and PLIC_L compared with duplication carriers, but no significant differences were found between carriers and noncarriers (24). Using the UK Biobank sample, we now report significant increased FA in ALIC_L, PLIC_R, and PLIC_L in deletion carriers compared with duplication carriers. Additionally, significant differences between deletion and NoCNV carriers are found in ALIC_L and PLIC_L. ALIC has been shown to be associated with emotion, decision making, cognition, and motivation (36), whereas PLIC is an important structure for motor and sensory pathways (36). In this study, we investigated the interaction between copy number and age in imaging and cognitive measures. FA age trajectories (Figure S12 in Supplement 1) show a possible CNV × age interaction in ALIC, BodyCC, and SpleniumCC, where FA increases with age in deletion carriers, and differs from the typical gradual reduction of FA with age (37). However, these interactions were not significant after multiple comparison correction. A younger group (from childhood until adulthood) would be needed to reliably investigate the impact of 15q11.2 BP2-BP2 CNVs on white matter development.

Current and previous results also showed similar effects (increased FA in deletion carriers compared with duplication carriers) in different portions of the cingulum; significant effects were found on Cing_CG_L in the Icelandic sample and on Cing_CG_R, Cing_HIP_R, and Cing_HIP_L in the UK Biobank sample. In the UK Biobank sample, duplication carriers show significant reduced FA in these three tracts compared with NoCNV carriers. The cingulum connects components of the limbic system, where different portions reflect distinct functions (38). The hippocampal portion is linked to learning and episodic memory. Conversely, deletion carriers show reduced FA and increased RD in the fornix, a major output tract of the hippocampus that is also implicated in memory function. Although not significant, deletion carriers in the Icelandic sample also showed a decrease in FA in this structure (Figure S5 in Supplement 1).

We found some divergence between our previous and current results. While deletion carriers showed increased FA in PTR_R and ILF_L in the Icelandic sample, they show reduced FA in the UK Biobank sample, with a significant FA reduction in PTR_R when compared with NoCNV carriers (Figure S5 in Supplement 1). Furthermore, when looking at effect sizes in all tracts (Figures S8–S11 in Supplement 1), an overall pattern of increased FA in deletion carriers in the Icelandic sample can be appreciated (the only exceptions being fornix and bilateral CST), whereas in the UK Biobank, the pattern is more heterogeneous. Divergent results could be explained by important differences between the two studies: the UK Biobank sample is considerably larger in size, increasing statistical power to detect true associations; it also represents a more genetically heterogeneous population of an older age than the Icelandic sample. Moreover, UK Biobank applied DTI acquisition protocols with higher resolution, which could also lead to more robust results.

NDDs have been generally associated with global decreases in FA (33,39,40), which contrasts with the findings of increased FA in 15q11.2 BP1-BP2 deletion carriers. This raises the question of how changes in FA relate to cognitive function and risk for disorder in these carriers. In our neuroimaging sample, deletion carriers performed worse during reaction time, fluid intelligence, symbol substitution, and trail making B tasks, whereas duplication carriers performed at a similar level as NoCNV control subjects in all tasks. The same pattern was observed when extending our analyses to all participants with cognitive data available, where additional effects in pairs matching and digit span tasks were seen in deletion carriers (Table S6 in Supplement 2). This pattern of effects is in line with previous studies, where the 15q11.2 BP1-BP2 deletion was reported as being more damaging than duplication for a variety of cognitive tests (7,16).

Our analyses show that FA in white matter tracts affected by CNV carrier status correlates positively with cognitive performance in reaction time, fluid intelligence, symbol substitution, and trail making B tasks. Mediation analysis revealed that changes in PTR_R partially mediated the effects of deletion in all cognitive tasks, where lower FA in PTR_R in deletion carriers contributed to 2%–6% of the CNV effect across tasks. PTR is known to connect the caudal parts of the thalamus to both the parietal and occipital lobes (41) and has been previously indicated as the strongest white matter predictor for fluid intelligence (42). Conversely, the increased FA seen in ALIC_L and Cing_HIP_L in deletion carriers had the opposite effect on cognition, removing part of the CNV effect on performance. These findings suggest that increased FA in these regions contributes to better cognitive performance in deletion carriers.

A recent study used data gathered through the Enhancing Neuro Imaging Genetics through Meta Analysis (ENIGMA) consortium (43) and UK Biobank to determine the effects of the 15q11.2 BP1-BP2 CNV on cortical and subcortical brain morphology. The study reported reduced brain surface area and thicker cortex in deletion carriers, where the significant differences in cortical thickness were more evident in the frontal, cingulate, and parietal lobes. Furthermore, this study found significant mediation effects of total surface area and cortical thickness on fluid intelligence, with similar proportions as the ones reported in this study (16). Taken together, these findings suggest that 15q11.2 BP1-BP2 CNV effects on white and gray matter provide partially complementary effects on cognitive ability.

Among the four genes in this region, *NIPA1* and *CYFIP1* are known to be involved in mechanisms that, when dysregulated, have the potential to alter white matter. *NIPA1* interacts with the bone morphogenic protein (BMP) receptor type II to inhibit BMP signaling, which contributes to axonal growth, guidance, and differentiation (44). Enhanced BMP signaling was found to cause abnormal distal axonal overgrowth at the presynaptic neuromuscular junction in a *Drosophila* model (45). *CYFIP1* is considered a likely contributor to 15q11.2 BP1-BP2–associated phenotypes. Dysregulations in this gene result in alterations in dendritic spine morphology and branching (46,47). CYFIP1 interacts in two distinct complexes (46)—the WAVE regulatory complex, which regulates actin remodeling during neural wiring (48), and the CYFIP1-eIF4E complex, which, through interactions with FMRP, regulates translation of FMRP-target messenger RNAs (49). FMRP is the gene product of *FMR1*, which, when mutated, causes fragile X syndrome, the most common monogenic form of intellectual disability (50).

In our previous study, we hypothesized that *CYFIP1* could be a primary contributor to white matter changes in 15q11.2 BP1-BP2 CNV carriers. Previous DTI studies have shown increased FA in patients with fragile X syndrome (51,52), similar to what we observed in 15q11.2 BP1-BP2 deletion carriers, suggesting that these changes could be in part due to disruptions in the CYFIP1-FMRP complex. Recently, we developed a novel *Cyfip1*-haploinsufficient rat line using CRISPR (clustered regularly interspaced short palindromic repeats)/Cas9 to assess the influence of *Cyfip1* on white matter (53). *Cyfip1* haploinsufficiency led to decreased FA, myelin thinning in the corpus callosum, and aberrant intracellular distribution of myelin basic protein in cultured oligodendrocytes. These findings contrasted with our previous results from the Icelandic sample, showing widespread increased FA in 15q11.2 BP1-BP2 deletion carriers. However, in the UK Biobank sample, reduced FA was found in PTR_R in deletion carriers, which contributed to worse cognitive performance. Decreased FA could indeed result from myelin deficits (54) that could be caused by dysregulations in *CYFIP1*, which could in turn affect cognition (55,56). Conversely, increased FA in ALIC_L and Cing_HIP_L led to better cognitive performance in deletion carriers, which could be associated with compensatory mechanisms as a response to primary deficits (e.g., in myelination or synapses). It is, however, difficult to speculate at this point, given that the individual or combined influence of the other three genes in this region on white matter is unknown, and disruptions to myelin and/or axons cannot be distinguished with traditional DTI methods.

The effect sizes reported here were overall smaller than the ones in the Icelandic sample. In this study, we compared carriers to thousands of control subjects, which provides a better estimate of the general population mean and therefore a more reliable estimate of effect sizes (57). In the Icelandic sample, carriers were compared with 19 NoCNV control subjects, which may have led to an overestimation of the CNV effect. The 15q11.2 BP1-BP2 deletion has been proposed as a variant of uncertain clinical significance. However, more recent meta-analyses on published case-control studies have classified the deletion as a pathogenic of mild effect size (58). It is also important to note that the recruitment in UK Biobank and Iceland relies on volunteers who put themselves forward to be scanned, which could result in a significant healthy volunteer bias (59).

Different sample characteristics and imaging acquisition protocols could explain some of the variability between our previous and current results. However, it is encouraging that we found concordant results from different samples, increasing our confidence of a CNV effect on white matter, particularly on the cingulum and internal capsule, which harbor important connections of the limbic system.

### Conclusions

We provide converging evidence from two independent samples with different genetic and environmental backgrounds supporting the effects of 15q11.2 BP1-BP2 carrier status on white matter microstructure, with larger effects in deletion than duplication carriers. Our results also point toward a dose-dependent effect, showing a linear trend in most tracts, where carriers of no CNVs sit between deletion and duplication carriers. We further show that changes in white matter partially mediate the association between CNV carrier status and cognitive performance. These results add to the evidence of white matter changes as being an intermediate phenotype between genetic risk variants and cognitive or clinical phenotypes.

## Supplementary Material

Supplement 2

Key Resources Table
