# Supplementary material for: Analysis of Diffusion Tensor Imaging Data From the UK Biobank Confirms Dosage Effect of 15q11.2 Copy Number Variation on White Matter and Shows Association With Cognition
Source: Biol Psychiatry. 2021 Sep 1;90(5):307–16. doi: 10.1016/j.biopsych.2021.02.969 (PMC8343146; doi:10.1016/j.biopsych.2021.02.969)
Supplement: Supplement 1 [file mmc1.pdf]

Analysis of Diffusion Tensor Imaging Data From the UK Biobank Confirms Dosage Effect of 15q11.2 Copy Number Variation on White Matter and Shows Association With Cognition

Supplement 1

Supplemental Methods

CNV quality control

The majority of participants in the UK Biobank (~450 000) were genotyped using the Affymetrix UK Biobank Axiom ® array (820 967 probes), with 50 000 participants initially genotyped using the Affymetrix UK BiLEVE Axiom array (807 411 probes). The two arrays were shown to be very similar (over 95% common content). The description of DNA extraction and processing workflow are further described at https://biobank.ctsu.ox.ac.uk/crystal/crystal/docs/genotyping_sample_workflow.pdf. Normal-ized signal intensity, genotype calls and confidence intervals were generated using ~750 000 biallelic markers that were further processed with PennCNV-Affy software (1). For quality control, individual samples were excluded if they had >30 CNVs, a waviness factor >0.03 or <-0.03, or a call rate <96%. Individual CNVs were also excluded if they were covered by <10 probes or had a density coverage of less than one probe per 20 000 base pairs (2).

Details on cognitive data processing

Cognitive data collected from UK Biobank participants were considered for analyses. When appropriate, data were transformed so that higher scores indicated higher performance.

The Pairs matching task tests episodic memory, where six pairs of cards were shown for three seconds to each participant, then turned over and the participant was asked to identify all correct pairs in as few tries as possible. We used the total number of errors made in round

2 (field 399) of the pairs matching test (field 100030) for analyses, excluding any individual who did not achieve 6 correct matches, in order to exclude participants who did not complete the test. Data were not normally distributed and a log+1 transformation was applied.

The reaction time task tests simple processing speed, where participants played 12 rounds of the card game 'Snap'. Here, two cards were shown at a time, and the participants had to click a button as fast as possible if the cards were the same. We used the mean time to correctly identify matches (field 20023) for analyses. Outlying scores (1500 ms) were excluded. Data were not normally distributed and a log transformation was applied.

The Fluid Intelligence Score tests reasoning and problem solving, where thirteen verbal and numerical reasoning questions were made, after which the participants had to complete as many questions as possible within 2 minutes. We used the number of correct answers (field 20016) for analyses. Data were normally distributed, so no transformation was made.

The Digit Span task tests working memory, where participants were shown a number and asked to recall this once the number had disappeared. The number became one digit longer in each round (total of 12 rounds). We used maximum number of digits correctly recalled (field 4282) for analyses. Data were normally distributed, so no transformation was made.

The Symbol Digit Substitution task tests complex processing speed, where participants were asked to play a code-breaking game, which consisted in matching numbers to a set of symbols within 2 minutes. We used the number of symbol digit matches made correctly (field 20159) for analyses. Outlying scores (0-1 and >36) were excluded. Data were normally distributed, after outlier exclusion, so no transformation was made.

The Trail Making A and B tasks test visual attention and consists of both a numeric (A) and an alphanumeric (B) part. Participants were asked to connect circles with numbers (trail A), and with alternating numbers and letters (trail B), in the correct ascending order. For both tests, we used the time to complete the task (trail A – field 20156, trail B – field 20157) for

analyses. Data were not normally distributed, and a log transformation was applied for both tasks.

Supplemental Findings

Age and sex interaction

We found significant interactions between copy-number and age on FA in BodyCC, SpleniumCC, ALIC_R, and ALIC_L (Table S8), however none of these survived correction for multiple comparisons. Age trajectory plots, shown in Figure S12, suggest that this interaction is driven by deletion carriers, who do not follow the same age trajectory as NoCNV- and duplication carriers in which FA progressively decreases with age. Instead, deletion carriers seem to maintain or increase FA values with age. This pattern does not extend to cognitive performance, where no interactions between copy-number and age were found (Table S8 and Figure S13). We found an interaction between copy-number and sex only on FA in ACR_L, Cing_CG_R and Cing_HIP_L (Table S9); these interactions did not survive correction for multiple comparisons. No significant interaction between copy-number and sex was observed regarding cognitive performance (Table S9).

Supplemental References

1. Wang K, Li M, Hadley D, Liu R, Glessner J, Grant SFA, et al. PennCNV: An integrated hidden Markov model designed for high-resolution copy number variation detection in whole-genome SNP genotyping data. Genome Res [Internet]. 2007 Nov [cited 2020 May 15];17(11):1665–74. Available from: https://www.ncbi.nlm.nih.gov/pmc/articles/PMC2045149/

2. Kendall KM, Rees E, Escott-Price V, Einon M, Thomas R, Hewitt J, et al. Cognitive Performance Among Carriers of Pathogenic Copy Number Variants: Analysis of 152,000 UK Biobank Subjects. Biological Psychiatry [Internet]. 2017 Jul 15 [cited 2020 May 15];82(2):103–10. Available from: https://www.biologicalpsychiatryjournal.com/article/S0006-3223(16)32711-1/abstract

Supplemental Figures

White matter abbreviations used: GenuCC, genu of the corpus callosum; BodyCC, body of the corpus callosum; SpleniumCC, splenium of the corpus callosum; CST_R, right corticospinal tract; CST_L, left corticospinal tract; ALIC_R, right anterior limb of the internal capsule; ALIC_L, left anterior limb of the internal capsule; PLIC_R, right posterior limb of the internal capsule; PLIC_L, left posterior limb of the internal capsule; ACR_R, right anterior corona radiata; ACR_L, left anterior corona radiata; SCR_R, right superior corona radiata; SCR_L, left superior corona radiata; PCR_R, right posterior corona radiata; PCR_L, left posterior corona radiata; PTR_R, right posterior thalamic radiation; PTR_L, left posterior thalamic radiation; SStratum_R, right sagittal stratum; SStratum_L, left sagittal stratum; ExtC_R, right external capsule; ExtC_L, left external capsule; Cing_CG_R, right cingulum (cingulate gyrus portion); Cing_CG_L, left cingulum (cingulate gyrus portion); Cing_HIP_R, right cingulum (hippocampus region); Cing_HIP_L, left cingulum (hippocampus region); SLF_R right superior longitudinal fasciculus; SLF_L left superior longitudinal fasciculus; Unc_R, right uncinate fasciculus; Unc_L, left uncinate fasciculus

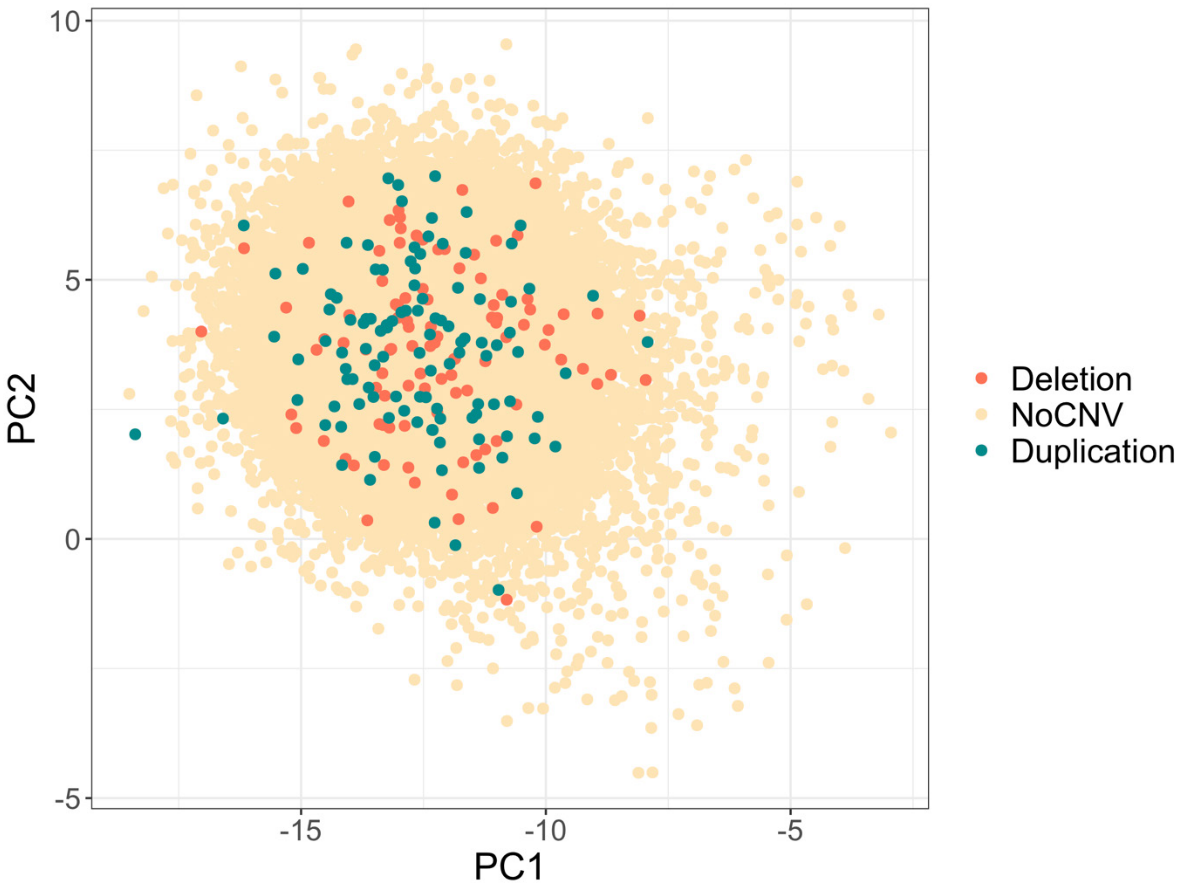

Figure S1 – Principal component 1 (PC1) vs Principal component 2 (PC2) showing population structure within the UK Biobank imaging sample used in this study (n=102 15q11.2 BP1-BP2 deletion carriers, n=113 duplication carriers, n=28,951 with no pathogenic CNVs (NoCNV)).

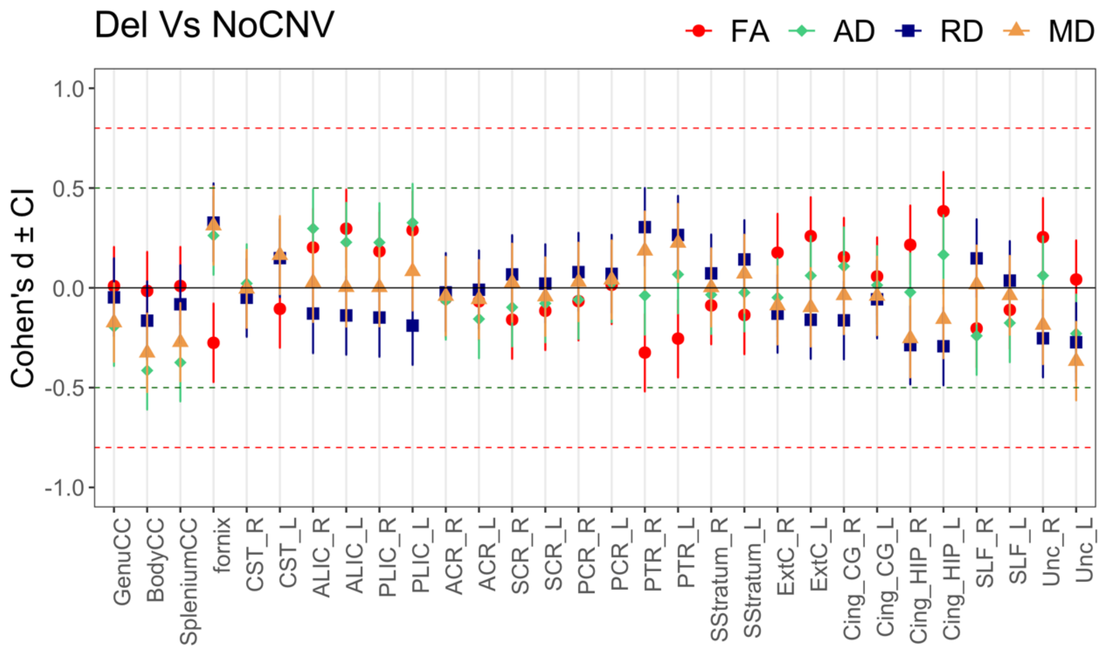

Figure S2 – Plots showing Cohen's d effect sizes for the contrast between deletion (Del) carriers and with no pathogenic CNVs (NoCNV) carriers for fractional anisotropy (FA), axial diffusivity (AD), radial diffusivity (RD) and mean diffusivity (MD).

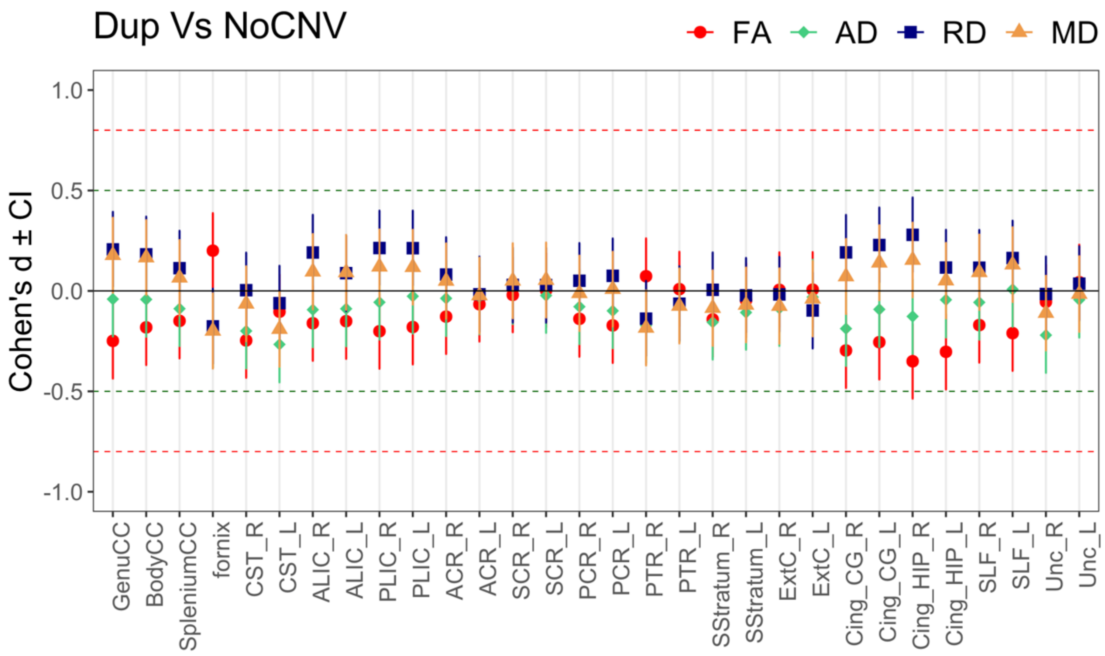

Figure S3 – Plots showing Cohen's d effect sizes for the contrast between duplication (Dup) carriers and with no pathogenic CNVs (NoCNV) carriers for fractional anisotropy (FA), axial diffusivity (AD), radial diffusivity (RD) and mean diffusivity (MD).

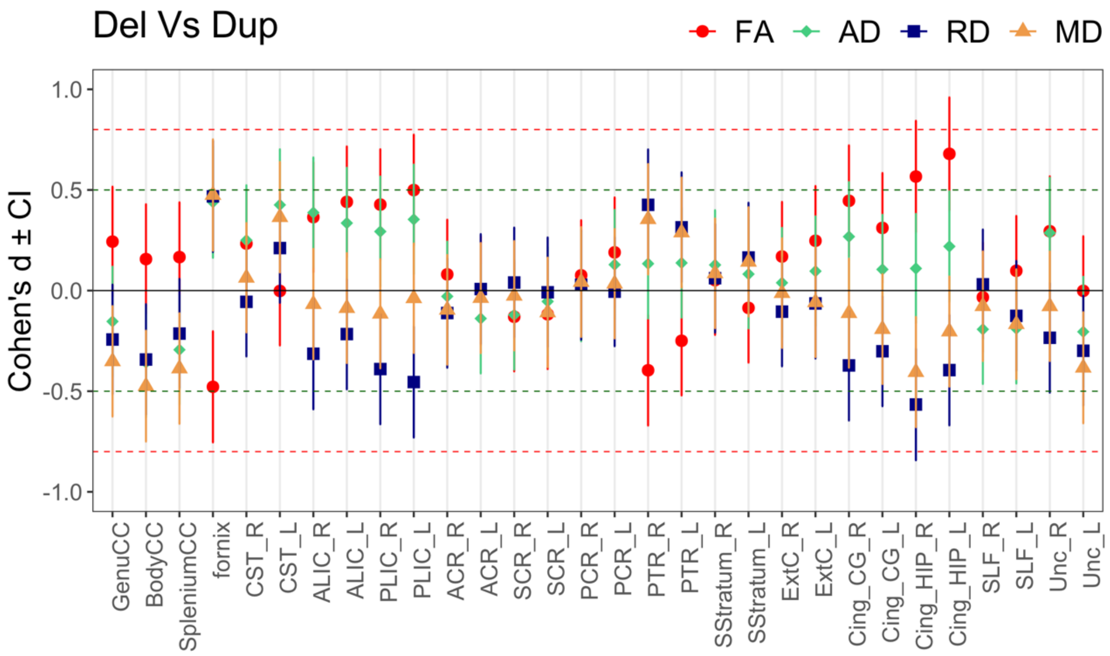

Figure S4 – Plots showing Cohen's d effect sizes for the contrast between deletion (Del) carriers and duplication (Dup) carriers for fractional anisotropy (FA), axial diffusivity (AD), radial diffusivity (RD) and mean diffusivity (MD).

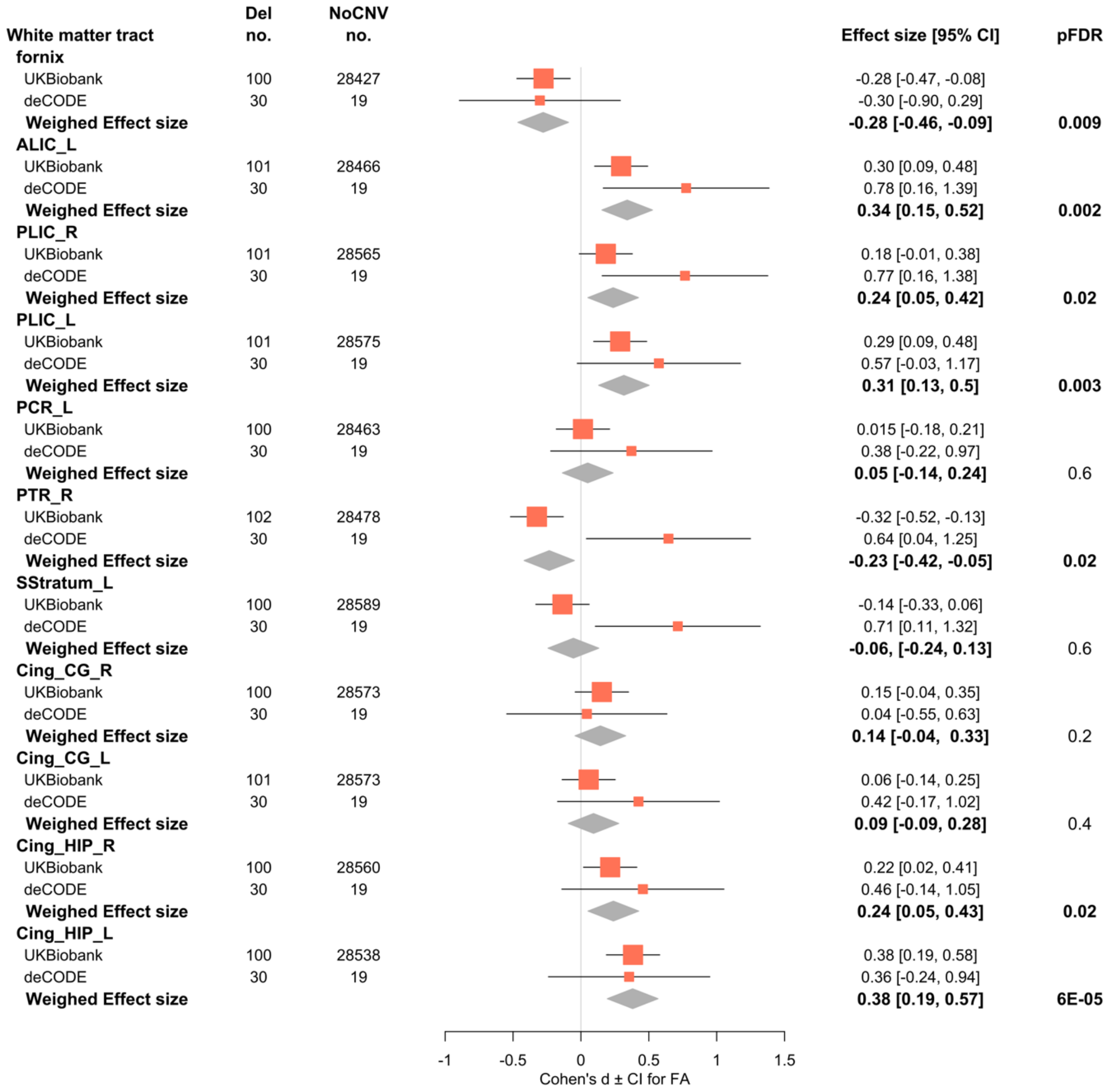

Figure S5 – Forest plot showing the Cohen's d effect sizes both in the UK Biobank and in the Icelandic samples, for the contrast between deletion (Del) carriers and with no pathogenic CNVs (NoCNV) carriers on fractional anisotropy. Here, only white matter tracts showing group effects in the UKBiobank sample and/or in the Icelandic sample are shown. The summary diamond shows the weighed effect size of both studies, calculated using the inverse of the variance method. pFDR, p value after false discovery rate correction.

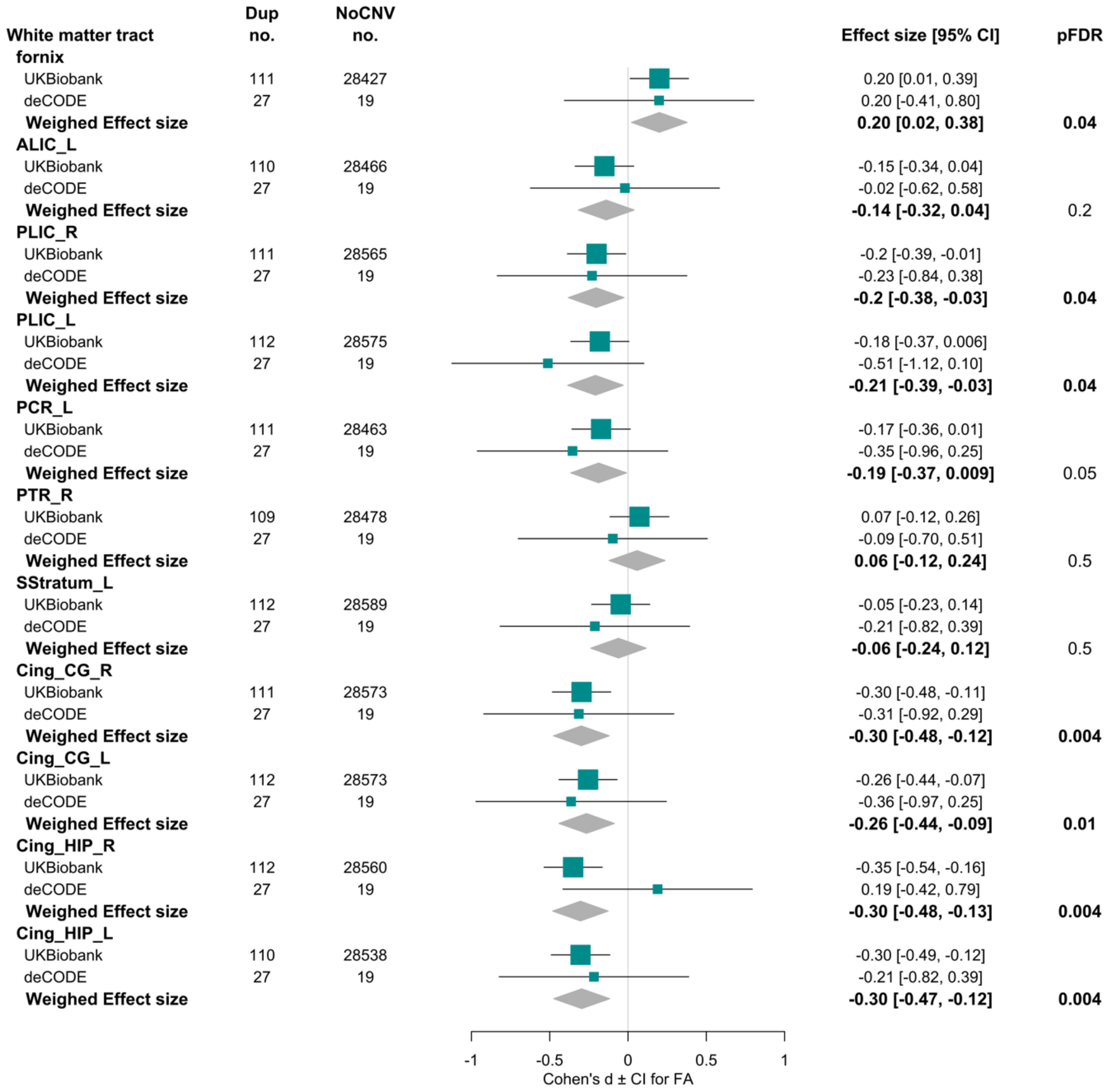

Figure S6 – Forest plot showing the Cohen's d effect sizes both in the UK Biobank and in the Icelandic samples, for the contrast between duplication (Dup) carriers and with no pathogenic CNVs (NoCNV) carriers on fractional anisotropy. Here, only white matter tracts showing group effects in the UKBiobank sample and/or in the Icelandic sample are shown. The summary diamond shows the weighed effect size of both studies, calculated using the inverse of the variance method. p_{FDR}, p value after false discovery rate correction.

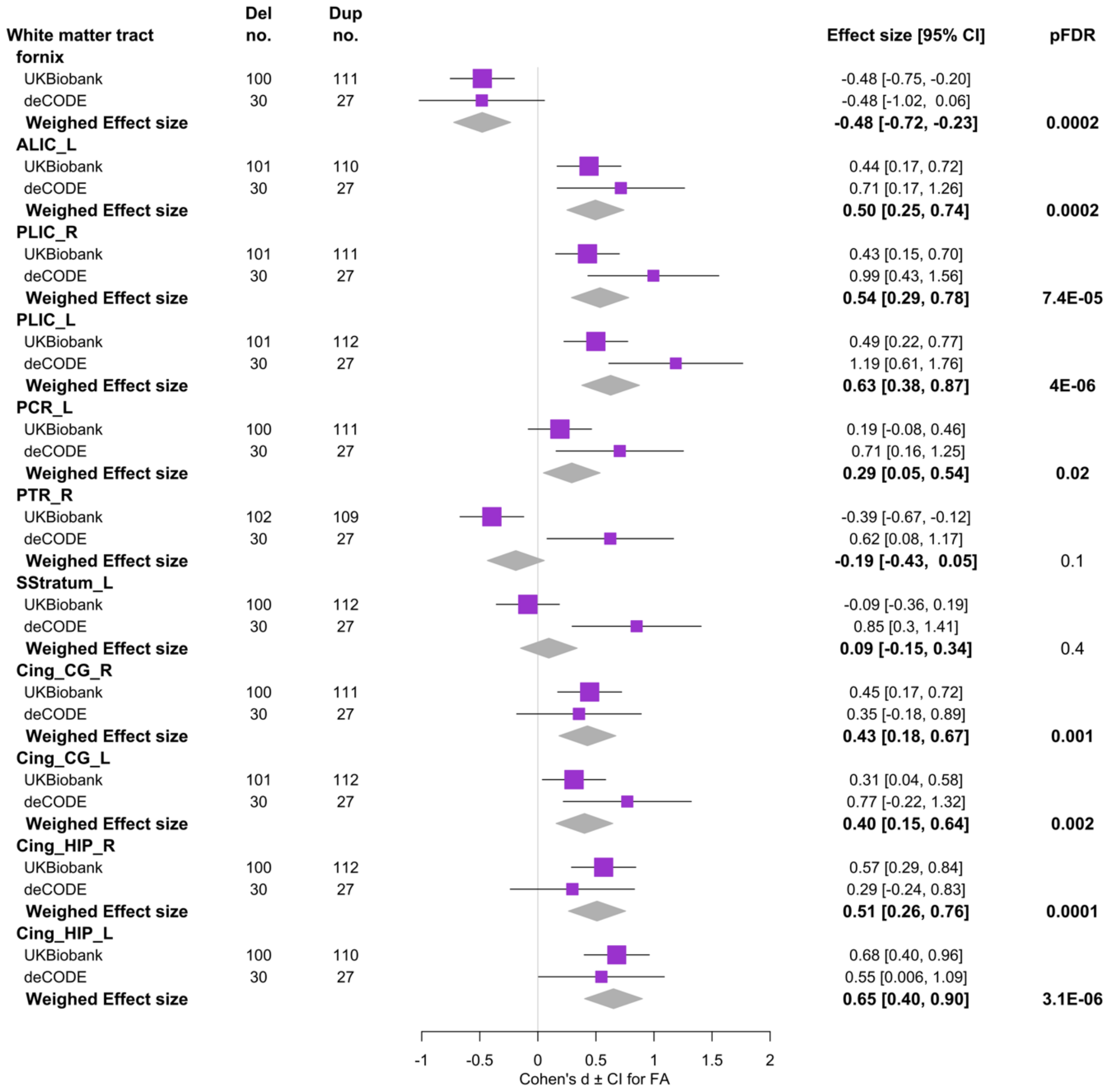

Figure S7 – Forest plot showing the Cohen's d effect sizes both in the UK Biobank and in the Icelandic samples, for the contrast between deletion (Del) carriers duplication (Dup) carriers on fractional anisotropy. Here, only white matter tracts showing group effects in the UKBiobank sample and/or in the Icelandic sample are shown. The summary diamond shows the weighed effect size of both studies, calculated using the inverse of the variance method. p_{FDR}, p value after false discovery rate correction.

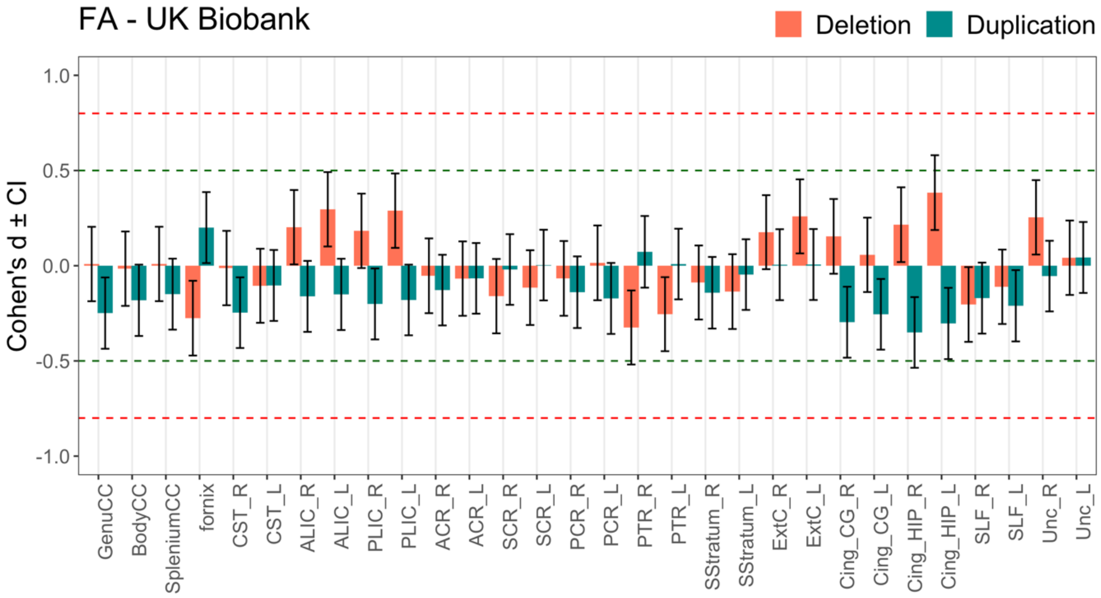

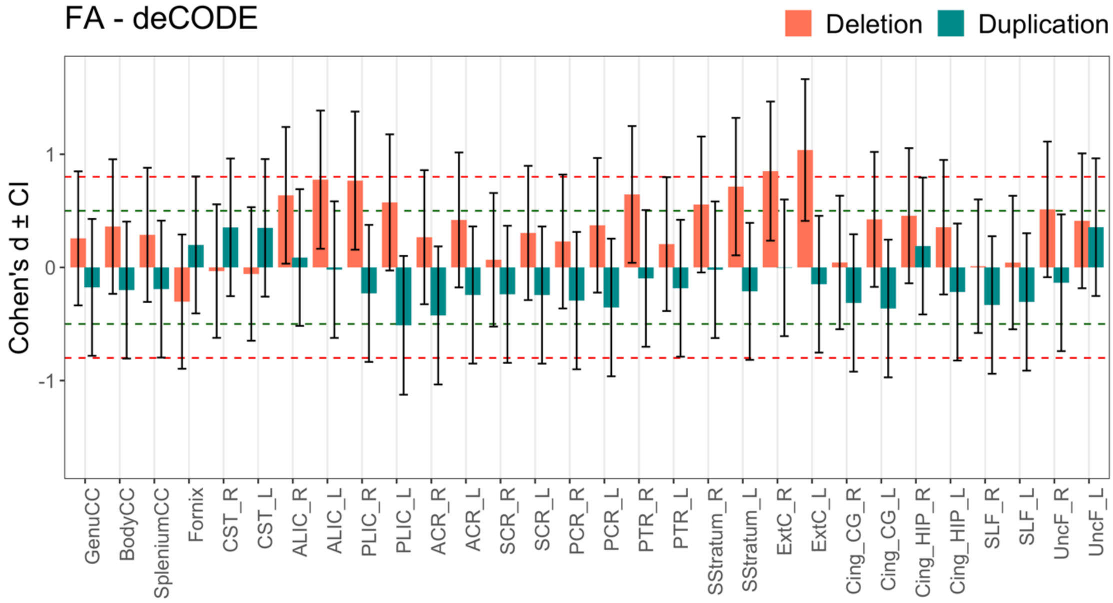

Figure S8 – Diverging bars showing Cohen’s d effect sizes for fractional anisotropy in the UK Biobank sample (top) and in the Icelandic sample (bottom), for the 30 white matter tracts considered in the study. In the UK Biobank sample, 102 deletion carriers and 113 duplication carriers were compared to 28,951 with no pathogenic CNVs (NoCNV) carriers. In the Icelandic sample, 30 deletion carriers and 27 duplication carriers were compared to 19 NoCNV carriers. The thresholds where effects sizes are considered large (0.8) or medium (0.5), according to Cohen’s criteria, are represented by a vertical red or green dashed line, respectively.

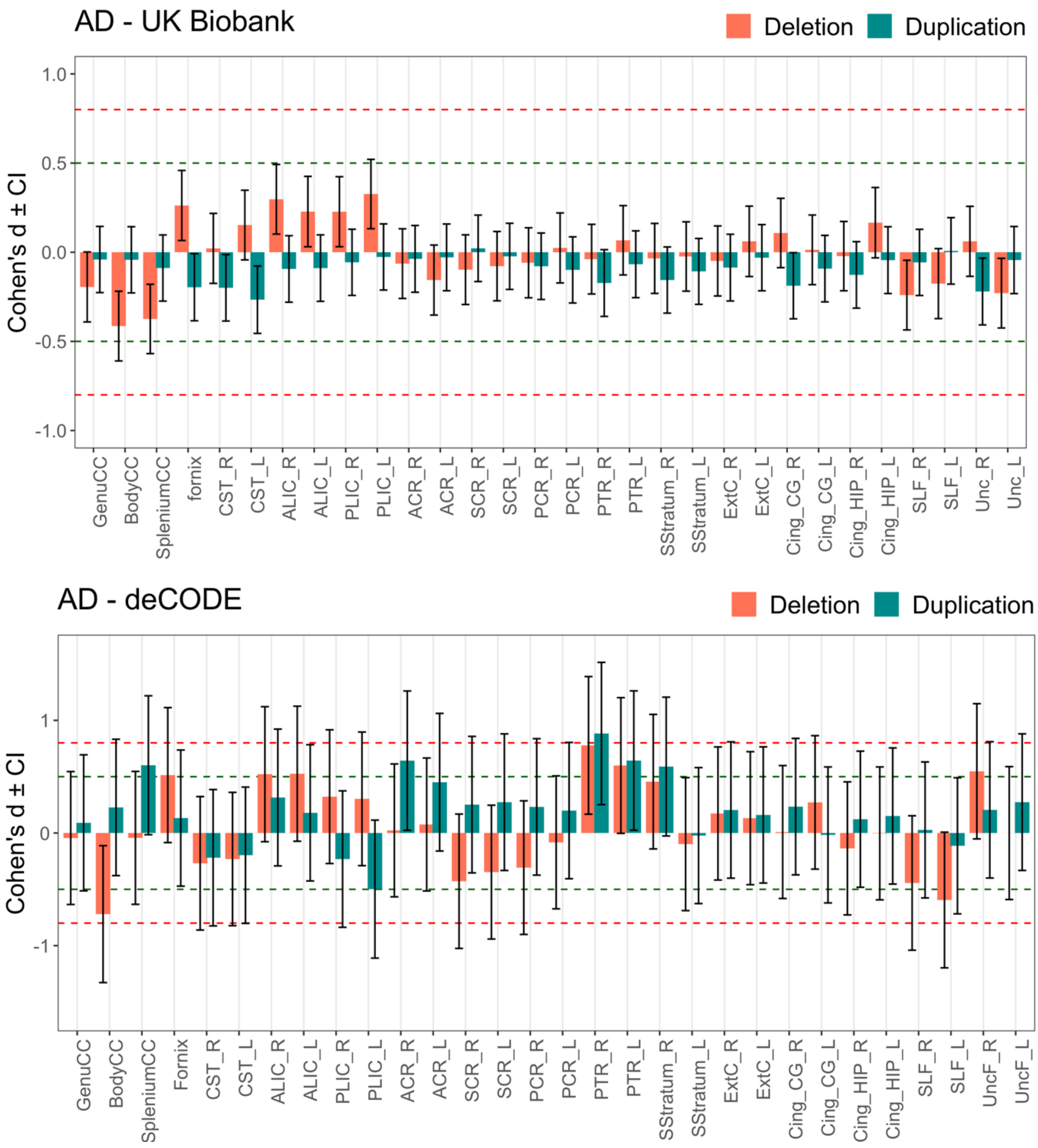

Figure S9 – Diverging bars showing Cohen's d effect sizes for axial diffusivity in the UK Biobank sample (top) and in the Icelandic sample (bottom), for the 30 white matter tracts considered in the study. In the UK Biobank sample, 102 deletion carriers and 113 duplication carriers were compared to 28,951 with no pathogenic CNVs (NoCNV) carriers. In the Icelandic sample, 30 deletion carriers and 27 duplication carriers were compared to 19 NoCNV carriers. The thresholds where effects sizes are considered large (0.8) or medium (0.5), according to Cohen's criteria, are represented by a vertical red or green dashed line, respectively.

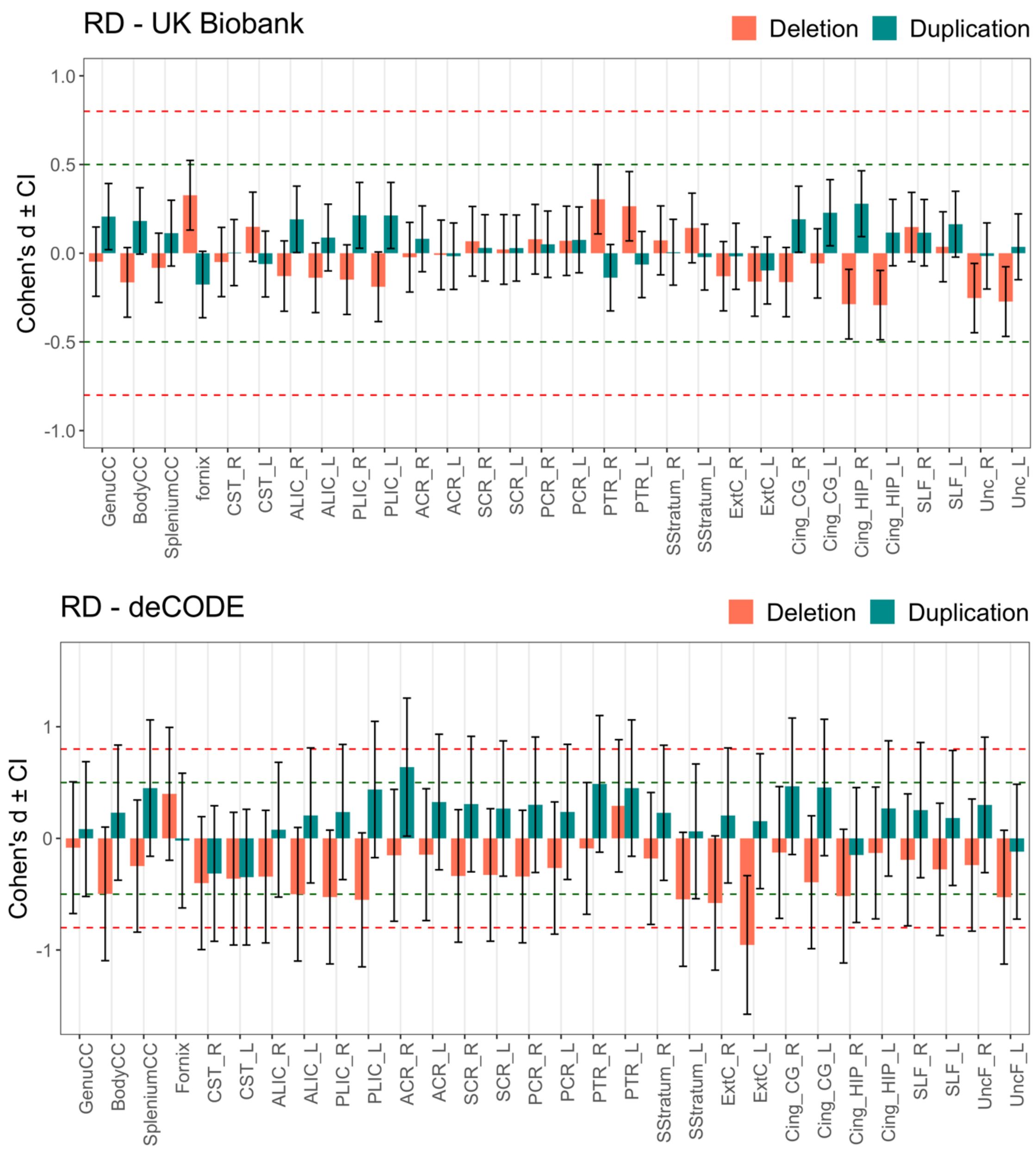

Figure S10 - Diverging bars showing Cohen's d effect sizes for radial diffusivity in the UK Biobank sample (top) and in the Icelandic sample (bottom), for the 30 white matter tracts considered in the study. In the UK Biobank sample, 102 deletion carriers and 113 duplication carriers were compared to 28,951 with no pathogenic CNVs (NoCNV) carriers. In the Icelandic sample, 30 deletion carriers and 27 duplication carriers were compared to 19 NoCNV carriers. The thresholds where effects sizes are considered large (0.8) or medium (0.5), according to Cohen's criteria, are represented by a vertical red or green dashed line, respectively.

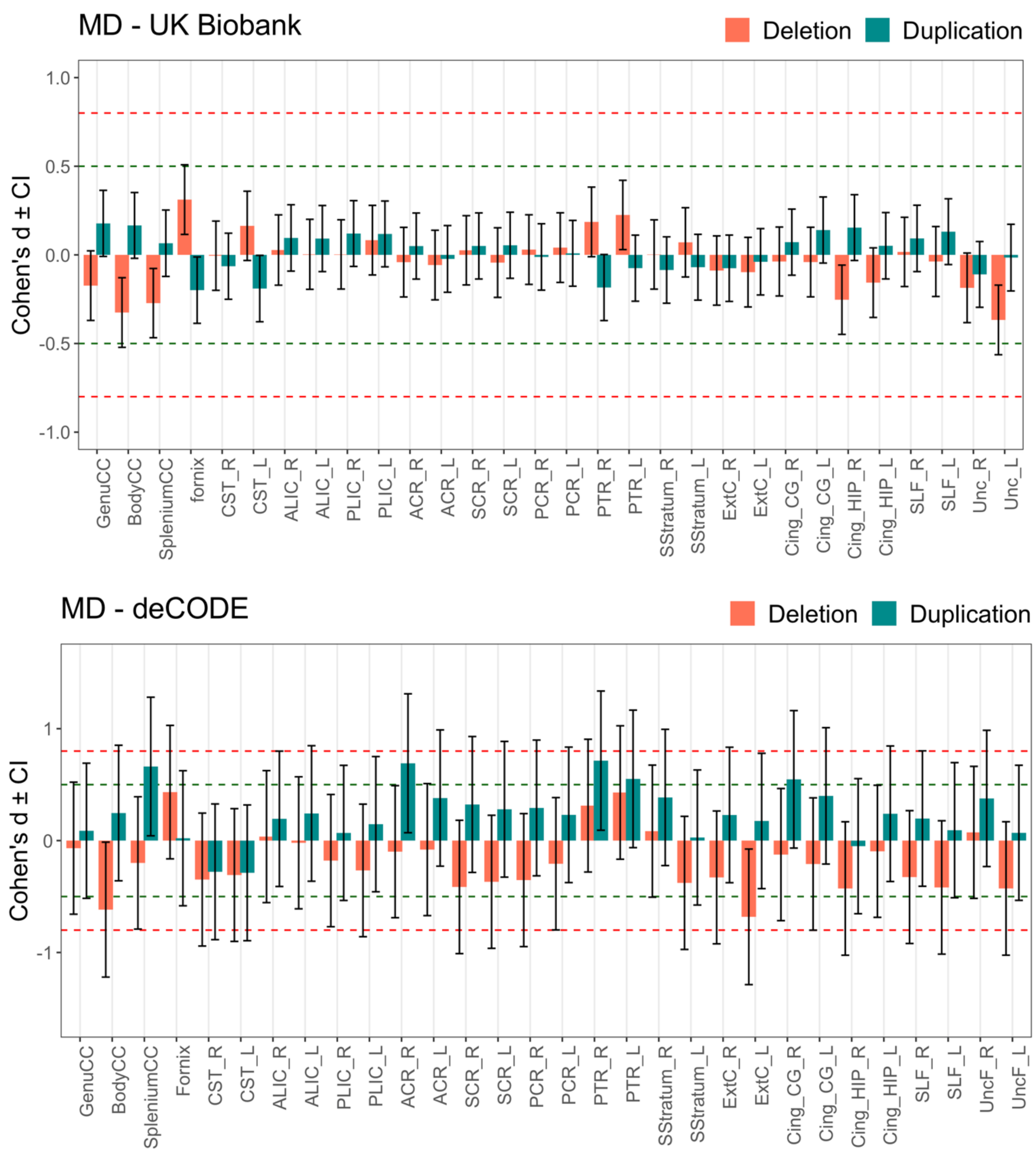

Figure S11 - Diverging bars showing Cohen's d effect sizes for mean diffusivity in the UK Biobank sample (top) and in the Icelandic sample (bottom), for the 30 white matter tracts considered in the study. In the UK Biobank sample, 102 deletion carriers and 113 duplication carriers were compared to 28,951 with no pathogenic CNVs (NoCNV) carriers. In the Icelandic sample, 30 deletion carriers and 27 duplication carriers were compared to 19 NoCNV carriers. The thresholds where effects sizes are considered large (0.8) or medium (0.5), according to Cohen's criteria, are represented by a vertical red or green dashed line, respectively.

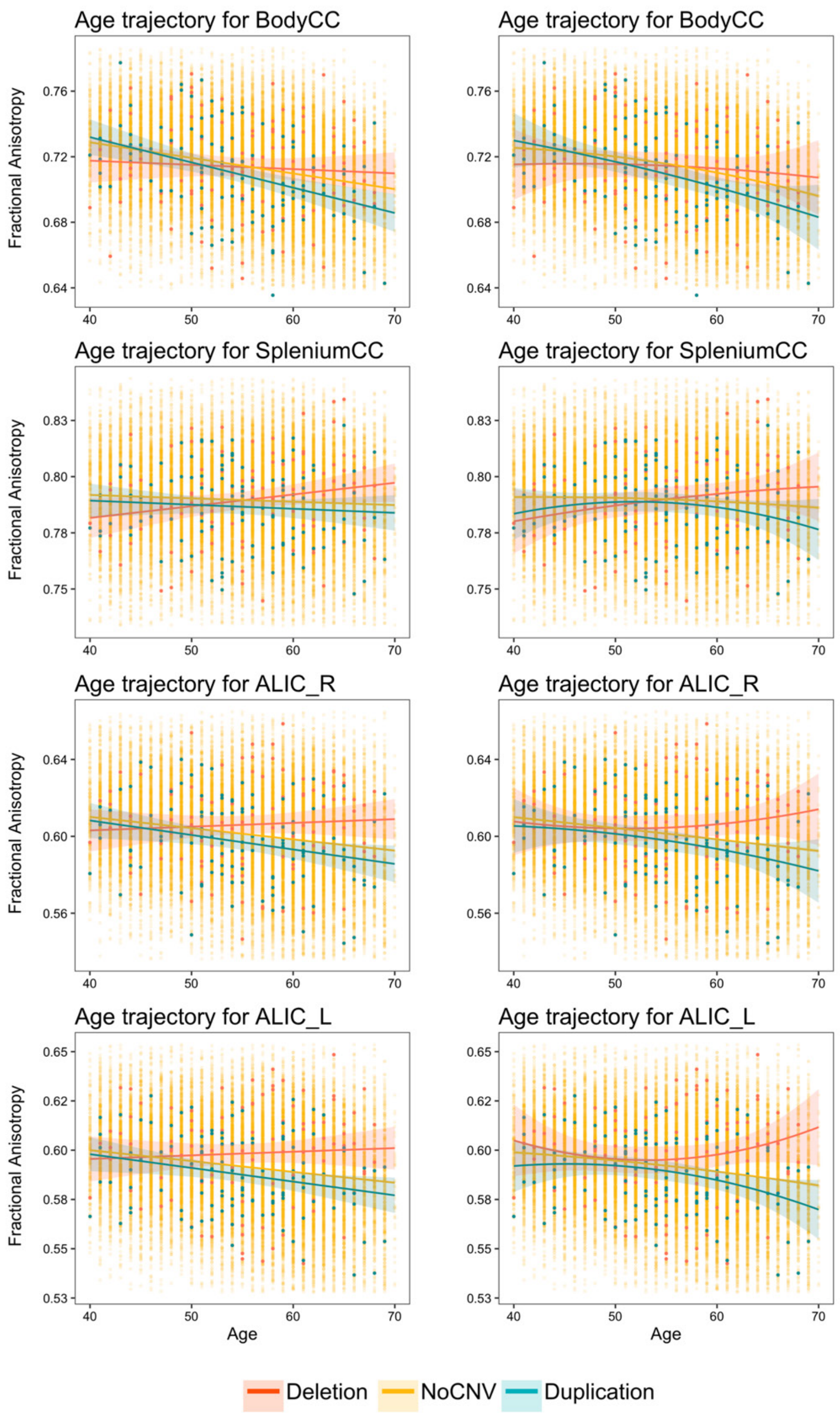

Figure S12 – Age trajectories showing the linear (left) and quadratic (right) relationship between fractional anisotropy and age. The three regression lines shown represent the three carrier groups (deletion, duplication and with no pathogenic CNVs (NoCNV) carriers), as indicated in the legend.

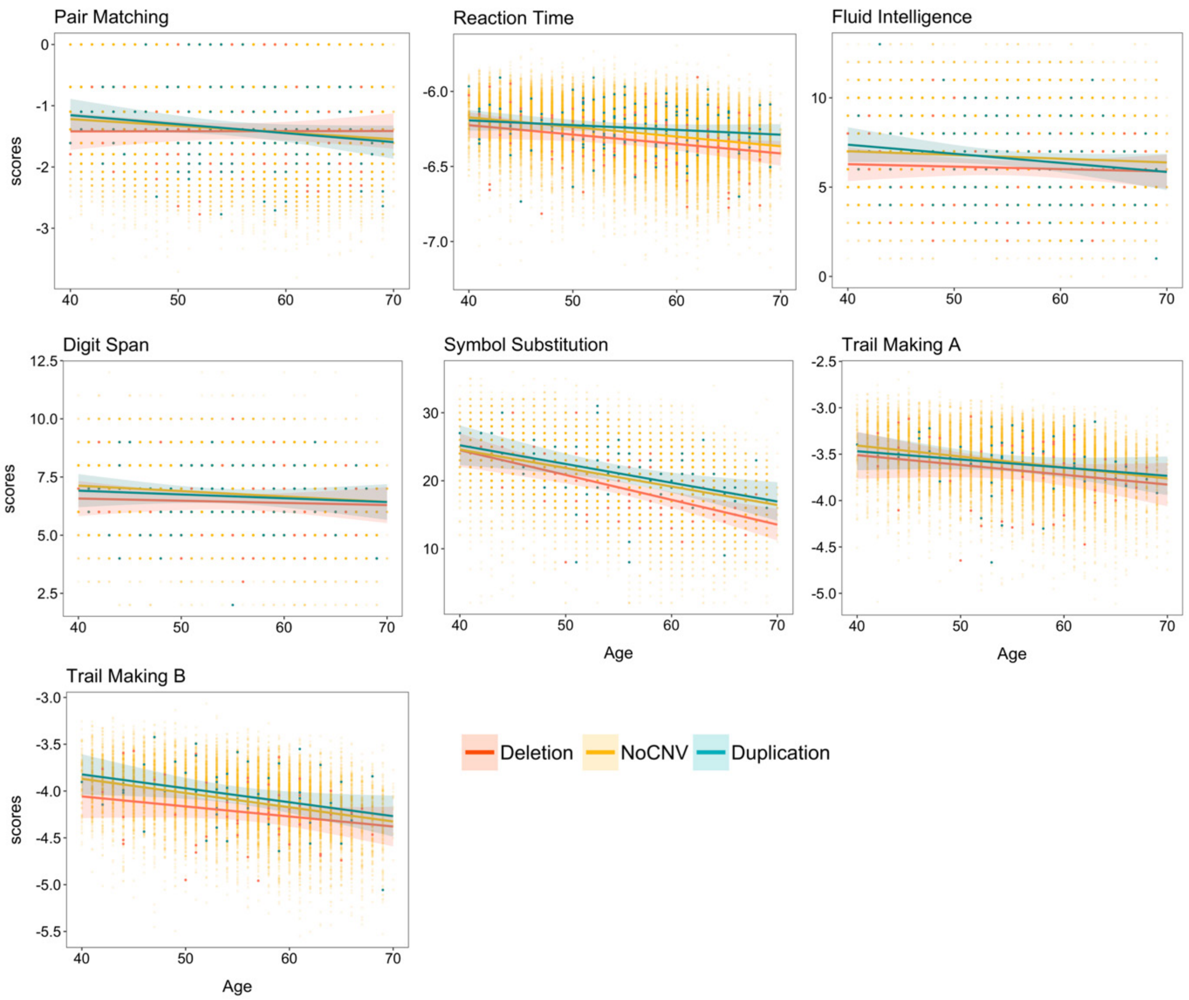

Figure S13 – Age trajectories showing the linear relationship between cognitive performance for each test and age. The three regression lines shown represent the three carrier groups (deletion, duplication and with no pathogenic CNVs (NoCNV) carriers), as indicated in the legend. All cognitive measures were transformed so that lower values represented poorer performance.

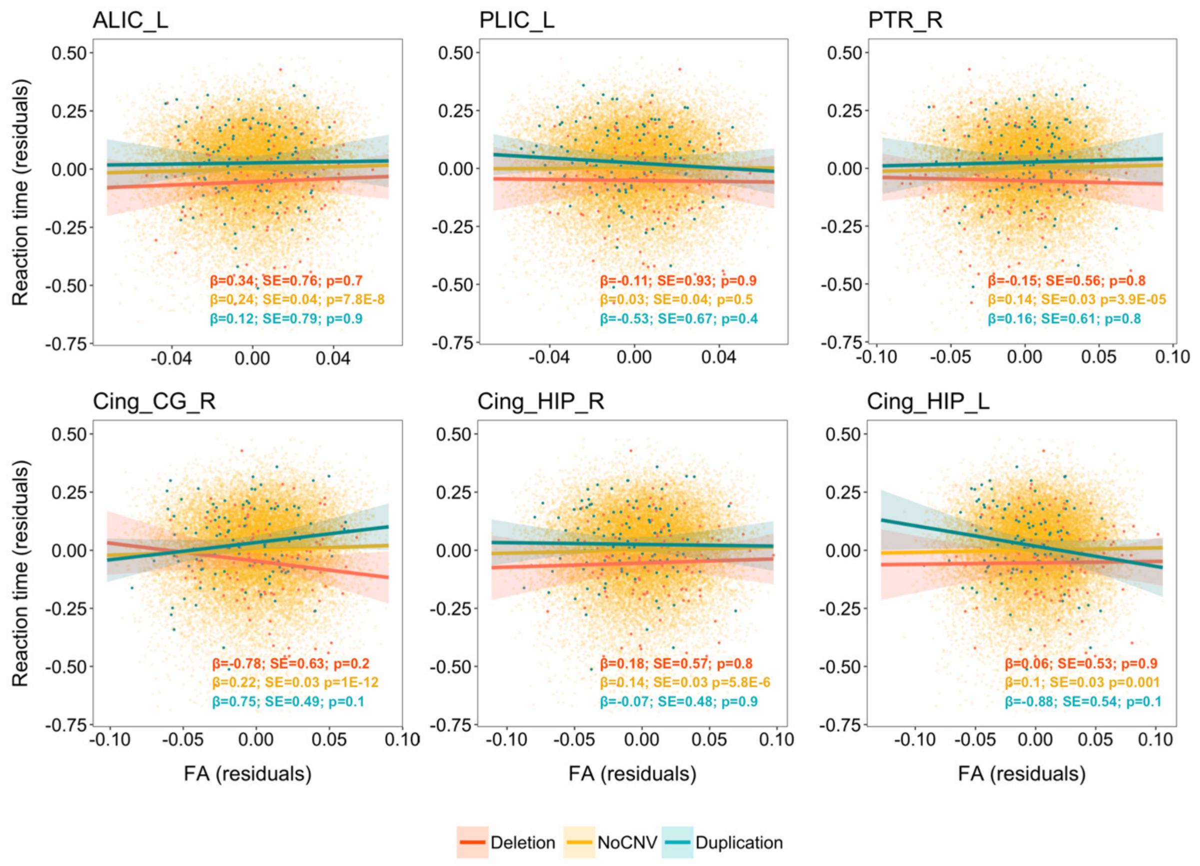

Figure S14 – Linear regression analyses comparing fractional anisotropy (FA) residual values to reaction time residual scores in the selected white matter tracts. Residuals were extracted from the linear regression taking age, sex and handedness as covariates for reaction time scores, as well as brain volume for FA measures. All cognitive measures were transformed so that lower values represented poorer performance. The three regression lines shown represent the three carrier groups (deletion, duplication and with no pathogenic CNVs (NoCNV) carriers), as indicated in the legend.

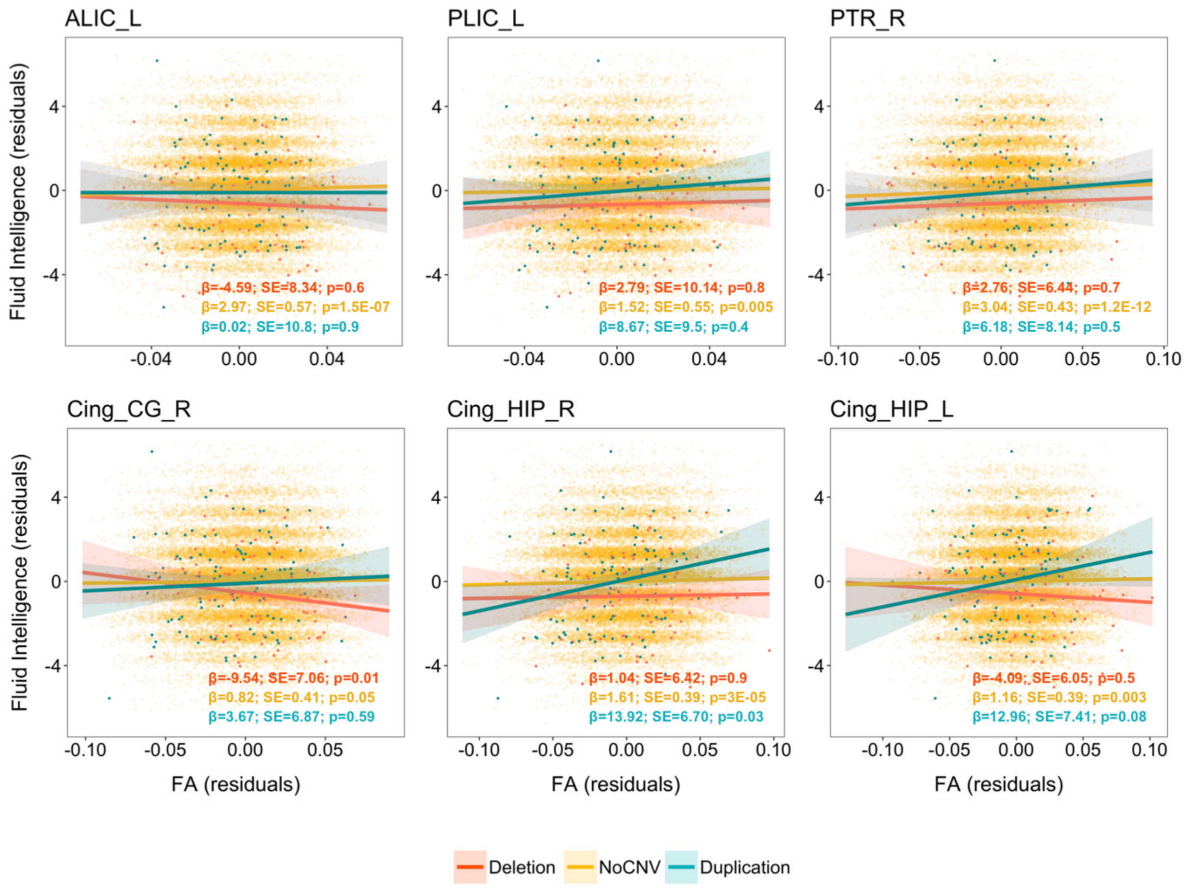

Figure S15 - Linear regression analyses comparing fractional anisotropy (FA) residual values to fluid intelligence residual scores in the selected white matter tracts. Residuals were extracted from the linear regression taking age, sex and handedness as covariates for fluid intelligence scores, as well as brain volume for FA measures. All cognitive measures were transformed so that lower values represented poorer performance. The three regression lines shown represent the three carrier groups (deletion, duplication and with no pathogenic CNVs (NoCNV) carriers), as indicated in the legend.

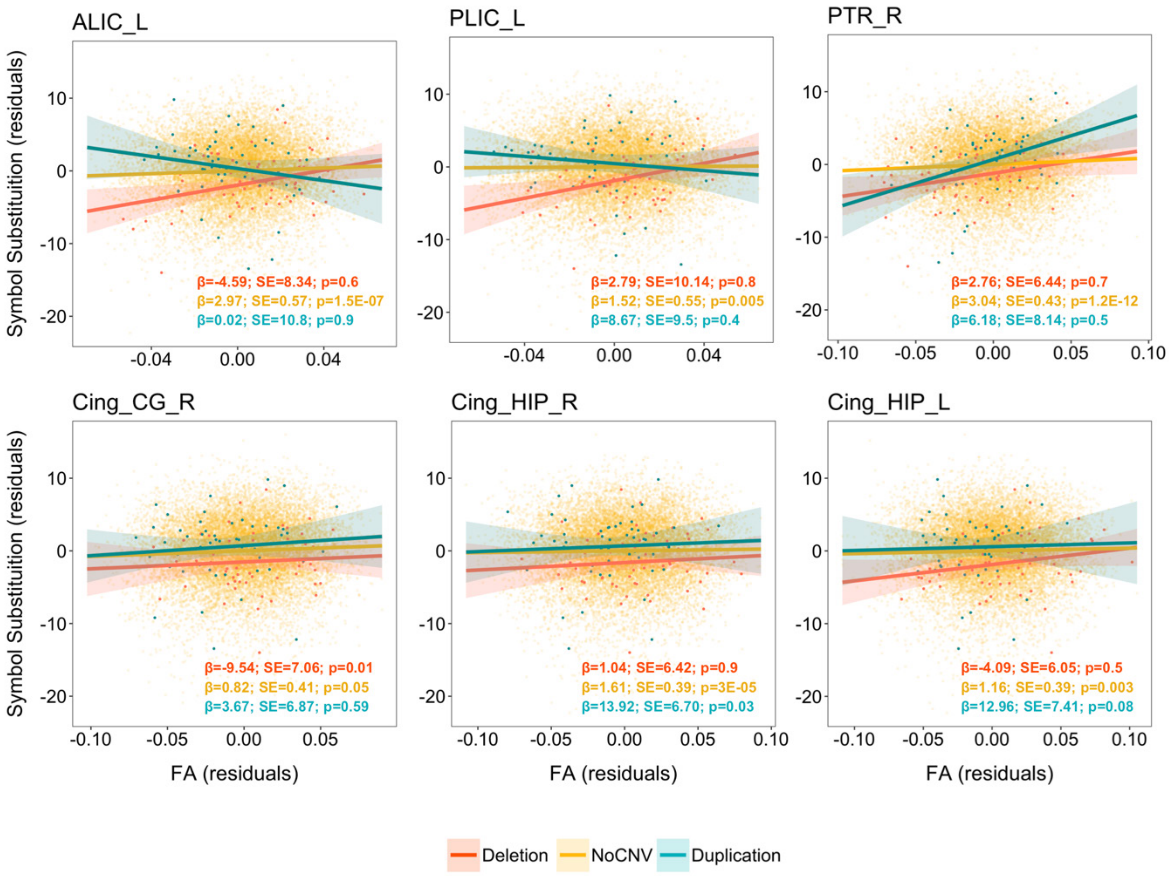

Figure S16 - Linear regression analyses comparing fractional anisotropy (FA) residual values to symbol substitution residual scores in the selected white matter tracts. Residuals were extracted from the linear regression taking age, sex and handedness as covariates for symbol substitution scores, as well as brain volume for FA measures. All cognitive measures were transformed so that lower values represented poorer performance. The three regression lines shown represent the three carrier groups (deletion, duplication and with no pathogenic CNVs (NoCNV) carriers), as indicated in the legend.

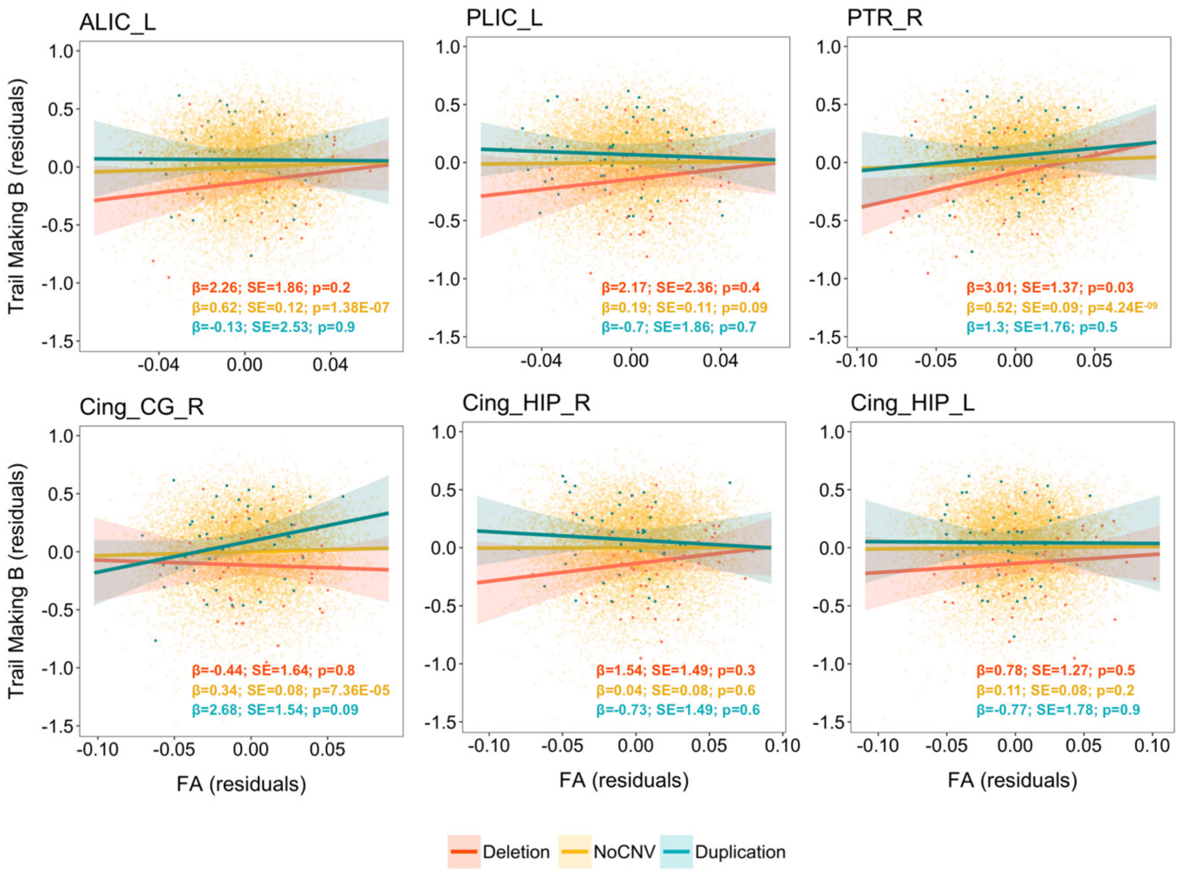

Figure S17 – Linear regression analyses comparing fractional anisotropy (FA) residual values to trail making B residual scores in the selected white matter tracts. Residuals were extracted from the linear regression taking age, sex and handedness as covariates for trail making B scores, as well as brain volume for FA measures. All cognitive measures were transformed so that lower values represented poorer performance. The three regression lines shown represent the three carrier groups (deletion, duplication and with no pathogenic CNVs (NoCNV) carriers), as indicated in the legend.
